# LDL receptor-related protein 1 (LRP1), a novel target for opening the blood-labyrinth barrier (BLB)

**DOI:** 10.1038/s41392-022-00995-z

**Published:** 2022-06-10

**Authors:** Xi Shi, Zihao Wang, Wei Ren, Long Chen, Cong Xu, Menghua Li, Shiyong Fan, Yuru Xu, Mengbing Chen, Fanjun Zheng, Wenyuan Zhang, Xinbo Zhou, Yue Zhang, Shiwei Qiu, Liyuan Wu, Peng Zhou, Xinze Lv, Tianyu Cui, Yuehua Qiao, Hui Zhao, Weiwei Guo, Wei Chen, Song Li, Wu Zhong, Jian Lin, Shiming Yang

**Affiliations:** 1 Department of Pharmacy, Peking University Third Hospital, Beijing, China; 2 Artificial Auditory Laboratory of Jiangsu Province, Xuzhou Medical University, Xuzhou, China; 3 National Engineering Research Center for the Emergency Drug, Beijing Institute of Pharmacology and Toxicology, Beijing, China; 4 College of Otolaryngology Head and Neck Surgery, Chinese PLA General Hospital, Beijing, China; 5 National Clinical Research Center for Otolaryngologic Diseases, Beijing, China; 6 Key Lab of Hearing Science, Ministry of Education, Beijing, China; 7 Beijing Key Lab of Hearing Impairment for Prevention and Treatment, Beijing, China; 8 Synthetic and Functional Biomolecules Center, Beijing National Laboratory for Molecular Sciences, Key Laboratory of Bioorganic Chemistry and Molecular Engineering of Ministry of Education, College of Chemistry and Molecular Engineering, Innovation Center for Genomics, Peking University, Beijing, China

**Keywords:** Target identification, Target validation, Molecular medicine

## Abstract

Inner ear disorders are a cluster of diseases that cause hearing loss in more than 1.5 billion people worldwide. However, the presence of the blood-labyrinth barrier (BLB) on the surface of the inner ear capillaries greatly hinders the effectiveness of systemic drugs for prevention and intervention due to the low permeability, which restricts the entry of most drug compounds from the bloodstream into the inner ear tissue. Here, we report the finding of a novel receptor, low-density lipoprotein receptor-related protein 1 (LRP1), that is expressed on the BLB, as a potential target for shuttling therapeutics across this barrier. As a proof-of-concept, we developed an LRP1-binding peptide, IETP2, and covalently conjugated a series of model small-molecule compounds to it, including potential drugs and imaging agents. All compounds were successfully delivered into the inner ear and inner ear lymph, indicating that targeting the receptor LRP1 is a promising strategy to enhance the permeability of the BLB. The discovery of the receptor LRP1 will illuminate developing strategies for crossing the BLB and for improving systemic drug delivery for inner ear disorders.

## Introduction

Inner ear disorders (IEDs) have a variety of consequences, including hearing loss (HL), vertigo, tinnitus, imbalance and lesions of the central auditory system. Currently, up to 1.5 billion people have hearing loss worldwide, and the number is expected to increase to 2.5 billion by 2050 according to the WHO.^1^ Despite the urgent need for IED treatment, options for IED are limited, and no therapeutic drugs can restore hearing. For patients with moderate and severe HL, hearing aids and cochlear implantation are standard protocols. However, for patients with mild symptoms, such as mild to moderate HL, autoimmune-related HL, age-related HL, noise-induced HL and sudden HL, periodic medication could be a more appropriate strategy. However, systemic drug administration is greatly limited in effectiveness by the blood-labyrinth barrier (BLB), which restricts the entry of drugs from the blood to the inner ear. The alternative of invasive local drug administration can cause damage and is ineffective in some patients.

The cochlea is a snail-shaped organ filled with fluid, with three and half turns in pigs and two and half turns in mice. Each turn is divided into three chambers, among which the scala vestibuli and scala tympani are filled with perilymph and the scala media is filled with endolymph. The scala media comprises the organ of Corti (OC) and stria vascularis (SV). In the OC, the sensory receptor cells are called hair cells (HC) and mechanoelectrically transduce sound into neuronal action potentials. Spiral ganglion neurons (SGNs) transduct the neural action potentials into the hearing cortex. In mammals, the number of sensory HCs is limited and cannot be regenerated when damaged. In SV, three layers of cells are present, namely, marginal cells (MCs), intermediate cells, and basal cells (BCs). MCs and BCs are connected to the same type of cell by tight junctions (TJs), and the space between the two types of cells is referred to as the intrastrial space. SV is responsible for ion exchange to maintain a high potential in the endolymph and low potential in the perilymph, which forms a potential gap that is essential for sensory HC transduction.

The BLB is a biological barrier between the vasculature and inner ear fluids, characterized by nonfenestrated capillaries and both TJs and gap junctions (GJs), and the entry of compounds usually decreases with increasing molecular weight.^2–4^ In the cochlea, the BLB consists of vascular endothelial cells (ECs) coupled with tight junctions (TJs). The vascular network comprises three parts: strial capillary beds in the SV, blood capillaries in the modiolus and blood capillaries in the basilar membrane (BM), which form the three important components of the BLB, the blood-strial barrier, the blood-nerve barrier and the blood-OC barrier, respectively. The intrastrial ECs, TJs, and GJs between marginal cells separate blood, intrastrial fluid and endolymph from each other, which further restricts the transportation of ions and other substances. The BLB is also reported to include large numbers of pericytes outside blood vessels in the basement membrane of the strial capillaries and spiral ligament and to include perivascular-resident macrophage-like melanocytes (PVM/Ms) and intermediate cells in the SV, which further restricts substance exchange.^5,6^ Although the overall structure is similar to that of the blood–brain barrier (BBB), the BLB is more complex and less permeable, which poses a greater challenge to the efficient delivery of therapeutics across the BLB.^2^

Therefore, noninvasive and targeted drug delivery to the inner ear via systemic administration shows poor inner ear permeability and tends to cause adverse effects due to off-target binding. Local delivery methods, including intratympanic, intracochlear and semicircular canal injection, can be damaging.^7–11^ Although drug carriers such as adeno-associated virus (AAV) and nanoparticle (NP)-based vectors have shown some therapeutic efficacy, most such strategies are also invasive.^10,12,13^ Hence, a strategy to efficiently cross the BLB for targeted inner ear delivery is urgently needed.

To address the above clinical obstacles, we aimed to find a target that could noninvasively open the BLB and deliver specific therapeutics into target cells in the cochlea. To achieve this goal, we analyzed the RNA sequencing data from different parts of the cochlea, aiming to discover specific membrane receptors that may facilitate the transport of drugs across the BLB. Among them, the receptor low-density lipoprotein receptor-related protein 1 (LRP1) was discovered. LRP1 has been reported to be expressed on the blood–brain barrier (BBB) and to be responsible for the transportation of multiple ligands across the BBB.^14–18^ In accordance with the similarity of the BLB and BBB, possibly transport molecules across the BLB. Adopting an in vivo phage-display-assisted biopanning strategy, we found an LRP1-binding peptide, IETP2, that can be transported to the inner ear. By targeting LRP1, IETP2 delivers multiple small molecules, including fluorescent dyes, antioxidation drugs and MRI contrast agents, to the inner ear, suggesting a universal strategy to cross the BLB by targeting LRP1.

## Results

### LRP1 is expressed and localized at the BLB interface and peripheral auditory cells

**Fig. 1:**
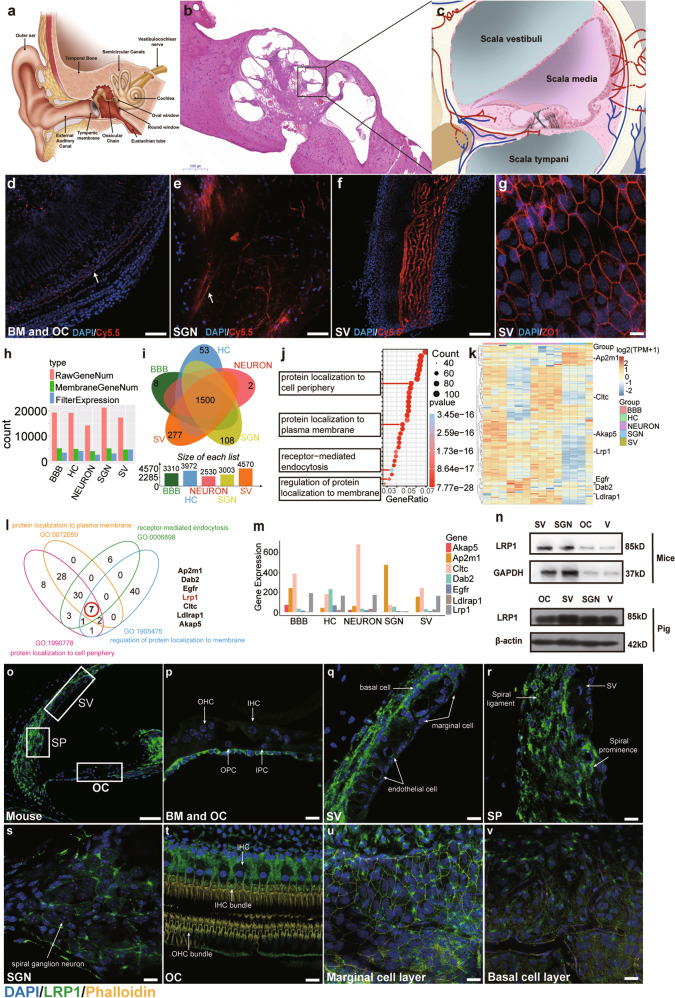
Expression and location of LRP1 in cochleae. **a** Schematic overview of the hearing system, consisting of the outer, middle and inner ears. The inner ear is divided into the cochlea and vestibular organ, which are in turn composed of the saccule, utricle and three semicircular canals. **b** Colloidin-embedded hematoxylin & eosin staining (CE-HE) image of the porcine cochlea. **c** Schematic representation of the microstructure in the black dotted rectangle in **b**. The cochlea consists of three and a half turns. It has 3 chambers called the scala media, which is filled with endolymph, and the scala tympani and scala vestibuli, both of which are filled with perilymph. **d**–**g** The BLB distribution in the mouse cochlea. **d** The spiral vessel in the BM (white arrow). **e** The blood vessel in the modiolus region (white arrow). **f** shows the capillaries in the SV. **g** shows the tight junctions between marginal cells. **h** Annotated gene expression in BBB, BLB (SGN and SV), hair cells (HC) and neuron cells (NEURON) from mRNA profiles. Red: all annotated genes. Green: membrane-related genes. Blue: filtered membrane-related genes. **i** VENN analysis showing the 1500 overlapping genes from the five groups. **j** GO annotation showing the cell components enriched among the 1500 overlapping genes annotated in **i**. **k** Heatmap showing the expression levels of all the genes from four GO subterms in **j** that were related to “protein localization to cell periphery”, “protein localization to plasma membrane”, “regulation of protein localization to membrane” and “receptor-mediated endocytosis. **l** VENN map showing 7 overlapping genes in the above four GO subterms in **k**, namely, *Ap2m1, Dab2, Egfr, Lrp1, Cltc, Ldlrap1*, and *Akap5*. **m** The expression levels of the 7 overlapping genes from **l**. **n** Western blot results showing that LRP1 was widely expressed in different parts of the pig and mouse cochleae. SV: stria vestibuli, OC: organ of Corti, V: Vestibular organ, SGN: spiral ganglion neurons. **o**–**v** The localization of LRP1 in the mouse cochlea. Tissue slices were stained with anti-LRP1 antibody. **o**–**s** Cross-sections and (**t**–**v)** whole-mount tissues of the C57BL/6J mouse cochlea. DAPI: blue; LRP1: green; phalloidin: yellow. Scale bars: **d**, **e**, **f** 50 µm, **o** 100 µm, others 10 µm.)

To obtain a universal hearing system delivery vehicle to cross the BLB (detailed structures of the hearing system and BLB are shown in Fig. 1a–g and Supplementary Fig. S1) and BBB, we analyzed high-throughput sequencing data to obtain mRNA expression profiles in the BLB (SV, spiral ganglion neurons), the BBB (cerebral vascular endothelial cell), and the target cells of interest, including the hair cells (HC) and neurons in the central nervous system (Fig. 1h, i), respectively. We found that more than 20,000 genes were expressed in these tissues (Fig. 1h, red column). We annotated more than 5000 membrane-related genes (Fig. 1h, green column) and further processed the genes with an expression data filter to remove the genes with inconsistent expression in each group (Fig. 1h, blue column). After data filtration, a total of 1500 overlapping membrane-related protein genes, which were coexpressed in the BLB, the BBB and the target cells of interest, were obtained by VENN analysis (Fig. 1i) and subjected to sequential GO analysis (Fig. 1j and Supplementary Table. S1), in which four subterms referred to as transmembrane transport (i.e., protein localization to cell periphery, protein localization to plasma membrane, regulation of protein localization to membrane, and receptor-mediated endocytosis) by GO annotation were selected (Supplementary Table. S2) and subjected to indiscriminate clustering (Fig. 1k). Subsequent VENN analysis revealed seven overlapping candidate genes (*Ap2m1, Dab2, Egfr, Lrp1, Cltc, Ldlrap1*, and *Akap5*) (Fig. 1l and Supplementary Table. S2), and the expression levels of these seven genes in different tissues are further indicated in Fig. 1m. Inspired by previous studies of LRP1 in the BBB and the predicted roles of LRP1 in the BLB,^19^ *Lrp1* was selected as the primary candidate gene for validation. LRP1 expression in mouse and pig vestibular organs (V), OC, SV, and SGNs (peripheral auditory system) was confirmed using Western blotting (Fig. 1n). The expression of LRP1 in mouse tissues was further verified with qRT–PCR (Supplementary Fig. S1G). LRP1 is highly conserved among species (>95% sequence homology between human and mouse or pig (sequences aligned in UniProt, data not shown), indicating that LRP1 is a universal receptor across species.

To further localize the specific cell types that expressed LRP1 receptor in mouse cochleae, immunostaining was performed with an anti-LRP1 antibody (Fig. 1o–v) and a previously reported LRP1 ligand Lix-FITC (Supplementary Fig. S2a–h). In the regions of BM and OC, LRP1 was localized in the basal layers of BM (Fig. 1o, p, t), Dieters’ cells, and inner and outer pillar cells (Supplementary Fig. S2i–k), and specifically, high LRP1 expression in IHCs and the hair bundles of HCs was observed (Fig. 1t and Supplementary Fig. S2 l). In the lateral wall, LRP1 localized to the spiral ligament (Fig. 1q, r), the basal cells of the SV (Fig. 1u and Supplementary Fig. S2h) and the endothelial cells of blood vessels (Fig. 1q). In the modiolus region, LRP1 was mainly localized in ganglion neurons, endothelial cells and the limbus region (Fig. 1s and Supplementary Fig. S2f). In the pig cochlea, the LRP1 receptor was localized in the same region as in the mouse cochlea (Supplementary Fig. S2m–r). In the regions of BM and OC, LRP1 was localized in the basal layers of BM and IHCs. In SV, LRP1 was mainly localized in basal cells, marginal cells and endothelial cells of the blood capillary. Overall, LRP1 was proven to be widely expressed in both mouse and pig cochlear cells, suggesting a potential role of LRP1 in crossing the BLB.

### Identification and validation of inner ear-targeting peptides (IETPs)

**Fig. 2:**
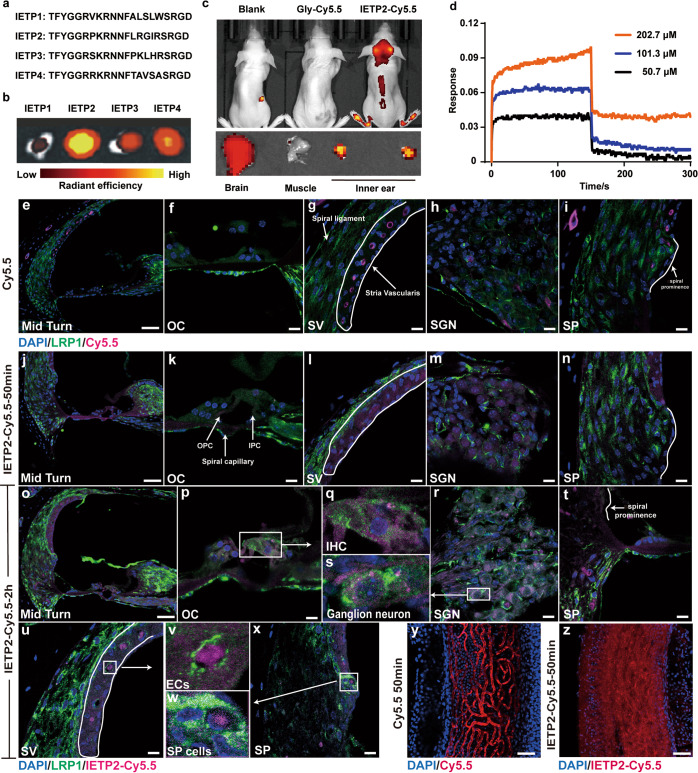
Identification of inner ear-targeting peptides and validation of the ability to cross the BLB in vivo. **a** Amino acid sequences of the four candidate IETP peptides. **b** Ex vivo fluorescence imaging showing the uptake of the four IETP peptides in mouse cochleae. **c** In vivo imaging showing the accumulation of IETP2-Cy5.5 in mouse cochleae. Gly-Cy5.5 served as a negative control. **d** Kinetic analysis of IETP2 binding to LRP1. A dissociation constant (K_D_) of 738 nM was obtained. **e**–**i** Immunofluorescence imaging of the mouse cochleae injected with Cy5.5. **j**–**n** Immunofluorescence imaging of mouse cochleae 50 min after the injection of IETP2-Cy5.5. Different regions (midturn, OC, SV, SGN and SP) were displayed, and immunostaining with anti-LRP1 antibody was performed. White arrows indicate regions of interest. **o**–**x** Immunofluorescence imaging of mouse cochleae 2 h after injection of IETP2-Cy5.5. **q**, **s**, **v**, **w** Zoomed-in immunofluorescence images of OC, SGN, SV, and SP (white rectangular area) in the mouse inner ear showing the colocalization of the receptor LRP1 and IETP2-Cy5.5 in IHCs, neurons, ECs and SP regions. **y**–**z** Microscopy images of the mouse cochleae SV injected with Cy5.5 or IETP2-Cy5.5 from a different perspective. Diffuse of IETP2-Cy5.5 into the intrastrial space was observed. DAPI: blue; LRP1: green; Cy5.5 and IETP2-Cy5.5: magenta or red. Scale bars: **e**, **j**, **o** 100 µm, **y**, **z** 50 µm, others 10 µm.)

LRP1 is a receptor with more than 40 natural ligands, and engineered peptide ligands have also been reported. A typical peptide ligand is ANG2,^20,21^ which has been reported to deliver paclitaxel to cross the blood–brain barrier for the treatment of metastatic breast cancer in clinical trials. However, improved peptide ligands are still needed, particularly improvements in the poor aqueous solubility, which impedes the drug injection of the peptide-drug conjugates. Herein, we selected a new LRP1 peptide ligand to assist drug delivery across the BLB. A phage-displayed random peptide library based on the ANG2 sequence was constructed. Amplified phages were intravenously injected into mice for mouse-based, phage-display-assisted in vivo biopanning. Mouse cochleae were then acquired, and phages within cochleae that showed the potential to cross the BLB were harvested and amplified for the next round of panning. After a total of four rounds of in vivo panning, four peptides with high rating scores, which we named inner ear-targeted peptides (IETPs), were selected for further validation (Fig. 2a).

To validate whether the selected IETPs could cross the BLB and to evaluate the inner ear-targeting efficacy, IETPs were covalently conjugated with the fluorescent dye Cy5.5 and intravenously injected into mice. In vivo and ex vivo fluorescence imaging of cochleae was used for evaluation. The fluorescence intensity of IETP2 in the cochleae was shown to be much higher than that of the other three peptides, suggesting that IETP2 might be the most efficient peptide for crossing the BLB (Fig. 2b). The IETP2 signal was visualized with in vivo fluorescence imaging (Fig. 2c). Compared to that in the brain and muscle, the signal of IETP2 detected in the cochleae was much stronger (Fig. 2c). We further validated the time-dependence of the accumulation of IETP2, and relatively long-term maintenance of IETP2 was observed in both the brain and cochleae (Supplementary Fig. S3a, b). Therefore, IETP2 was selected for the subsequent experiments.

### LRP1 binds IETP2 and may facilitate its transportation to the cochlea in vivo

To classify the roles of LRP1 in the transportation of IETP2 to the inner ear, the binding affinity of IETP2 to LRP1 was first determined using biolayer interferometry (BLI, Fig. 2d). A binding affinity of approximately 738 nM was calculated. In the in vivo experiments, mice were injected with either Cy5.5 or IETP2-Cy5.5. After sacrifice, the cochleae from the mice injected with free dye exhibited limited distribution inside blood vessels in the SV and SGN regions, with no signal in other substructures, such as BM, OC and SGN neuron cells (Fig. 2e–i). Fifty minutes post-injection, IETP2-Cy5.5 had been transported out of the cochlear capillaries and into some of the targeted cells, such as SGN cells, inner and outer pillar cells and all three layers of cells in SV (Fig. 2j–n and Supplementary Fig. S4a–e). SV was further imaged from another view. Retention of Cy5.5 in the blood vessel was clearly observed for mice SV receiving free Cy5.5, while considerable IETP2-Cy5.5 diffused to the intrastrial space (Fig. 2y, z). Two hours post-injection, the cochleae exhibited higher IETP2-Cy5.5 concentrations in hair cells, cells in the spiral prominence region (SP), SGN cells and nerve fibers (Fig. 2o–x and Supplementary Fig. S4f–t). Colocalization of the LRP1 receptor and IETP2-Cy5.5 could be partially observed, as shown in the zoomed-in images of white rectangles in Fig. 2o–x. The Manders’ colocalization coefficients (MCCs) of LRP1 and IETP2 in IHCs, ECs, SP cells, and SGNs were 0.71515, 0.64284, 0.56482, and 0.70552, respectively. The partial colocalization of LRP1 and IETP2 indicated that LRP1 may facilitate the transport of IETP2 in vivo.

To further test the generality of IETP2 in large mammals, IETP2 labeled with 5-TAMRA or Cy5.5 was injected into pigs via earlobe veins. Two hours post-injection, the pigs were sacrificed (Supplementary Figs. S5 and S6, representative images of three independent experiments). The fluorescence signals in the pig cochleae injected with dyes were retained in blood vessels in the SV and BM, and the spiral vessel could be clearly seen (Supplementary Figs. S5 and S6). The signal of dye-labeled IETP2 in the cochleae could be seen in the inner hair cells and supporting cells (Fig. S5d, e). Colocalization of the LRP1 receptor and IETP2 could be seen on the surface of IHCs. IETP2 was visualized in regions outside blood vessels in the SV (Fig. S5e), indicating that IETP2 could be transported into the inner ears of relatively large mammals.

### LRP1 is responsible for the endocytosis of IETP2

**Fig. 3:**
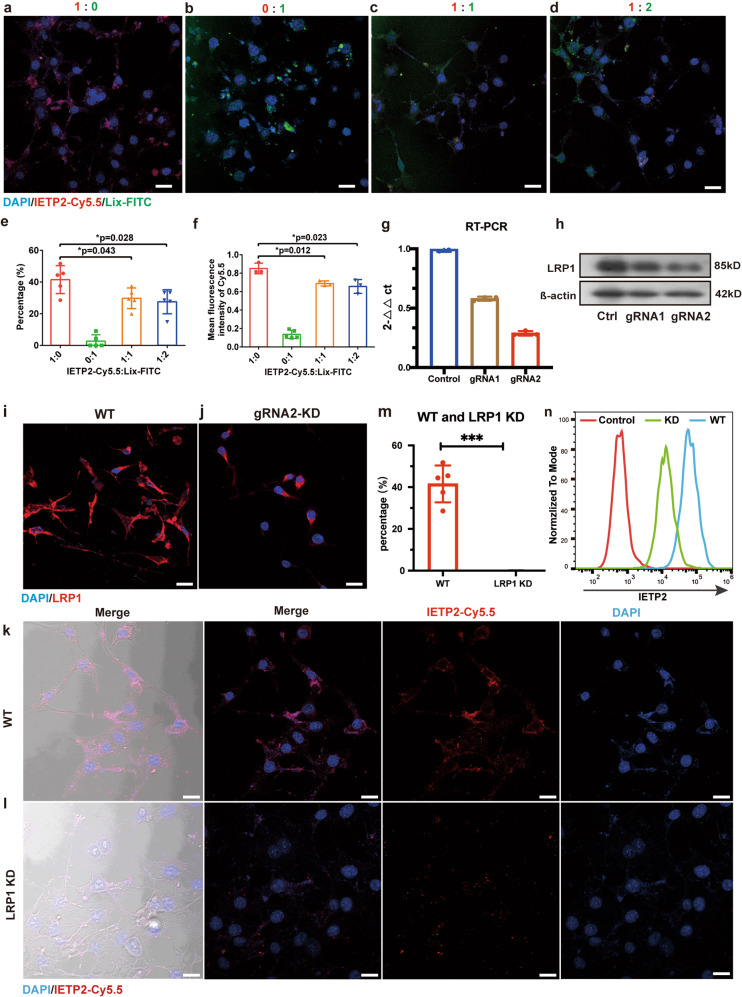
LRP1 is responsible for the endocytosis of IETP2. **a**–**d** Microscopy images showing the competing endocytosis of IETP2-Cy5.5 and Lix-FITC by HEI-OC1 cells. The endocytosis of IETP2-Cy5.5 was partially reduced by Lix-FITC. **e**-**f** Quantitative analysis of the fluorescence intensity in **a**–**d**. **e** Quantitative data showing the percentages of IETP2-Cy5.5-positive cells. **f** Quantitative data showing the mean intensity of IETP2-Cy5.5 in cells. **g** RT–PCR showing the knockdown (KD) efficiency of *Lrp1* with two different gRNAs. **h** Western blot showing the expression of LRP1 after LRP1 knockdown with two different gRNAs. **i**, **j** Immunofluorescence images showing the expression of LRP1 in WT and LRP1-KD HEI-OC1 cells. **k**, **l** Microscopy images showing the endocytosis of IETP2 in WT or LRP1-KD HIE-OC1 cells. Greatly reduced endocytosis of IETP2 in LRP1-KD HEI-OC1 cells was observed. **m** Quantitative analysis of the images in **k**, **l**. **n** Flow cytometry showing the reduced endocytosis of IETP2 in LRP1-KD HEI-OC1 cells. Error bars represent the mean ± SD. *N* = 5 for **e**, **f**, **m**. *N* = 3 for **g**. *p < 0.05, ***p < 0.001. Scale bars: 20 µm

The selected IETP2 peptide binds to LRP1 and can be internalized by LRP1-positive cells (Fig. 3). We further asked whether the endocytosis of IETP2 is involved in LRP1. To test this, a cell-based endocytosis competing assay was first performed. A previously reported LRP1 ligand, Lix-FITC (a polypeptide fused with ANG2 and labeled with fluorescein isothiocyanate), was allowed to compete with IETP2-Cy5.5 for endocytosis by HEI-OC1 cells. Microscopy showed that the endocytosis of IETP2 was partially reduced by the addition of Lix-FITC (Fig. 3a–d). Quantitative analysis of the imaging data showed the same trend (Fig. 3e, f), supporting the assumption that LRP1 is involved in the endocytosis of IETP2 by HEI-OC1 cells. In another antibody blocking assay, the endocytosis of IETP2 was also reduced by an anti-LRP1 antibody (Supplementary Fig. S7a), further supporting a critical role of LRP1 in the endocytosis of IETP2. In an in vivo assay, mixtures of Lix-FITC and IETP2-Cy5.5 were also intravenously injected into mice. The cochleae were collected 2 h post-injection, and tissue slices were imaged immediately. As shown in Fig. S7b, with increasing amounts of Lix-FITC, more IETP2-Cy5.5 was restricted in the blood vessels. Quantitative analysis showed that the signal intensity of intravascular IETP2-Cy5.5 was increased and that of perivascular IETP2-Cy5.5 was decreased with the addition of Lix-FITC (Supplementary Fig. S7c, d), indicating a competing relationship between IETP2 and Lix-FITC for binding to LRP1 and subsequent LRP1-mediated endocytosis.

To substantiate the critical role of LRP1 in the endocytosis of IETP2, an LRP1 knockout (or rather knockdown) assay was performed. LRP1 in HEI-OC1 cells was knocked out with CRISPR using two different sgRNAs packed in lentivirus (Supplementary Fig. S8).^22,23^ The efficacy of the two sgRNAs was primarily assessed by RT–PCR and Western blotting. The results showed that sgRNA2 was more efficient in knocking down LRP1 (Fig. 3g, h). The expression of LRP1 in wild-type (WT) HEI-OC1 and sgRNA2-induced knockdown HEI-OC1 cells (hereafter named LRP1-knockdown HEI-OC1) was further validated by immunofluorescence (Fig. 3i, j), which showed a notable reduction in LRP1 expression in LRP1-knockdown HEI-OC1 cells. The uptake of the peptide IETP2 by WT and LRP1-knockdown HEI-OC1 cells was evaluated by incubating either cell line with IETP2-Cy5.5. A significant reduction in peptide internalization in LRP1-knockdown HEI-OC1 cells was observed (Fig. 3k, l), indicating that LRP1 is essential for the entry of IETP2 into cells. The percentage of IETP2-positive cells was further quantitatively analyzed (Fig. 3m), which also showed the same reduction in LRP1-knockdown HEI-OC1 cells. Flow cytometry analysis of the endocytosis assay also proved that after LRP1 knockdown, the endocytosis of IETP2 was reduced (Fig. 3n). In brief, the above data support that the entry of IETP2 into cells is involved in and mediated by the receptor LRP1.

### IETP2 delivers different compounds across the BLB

**Fig. 4:**
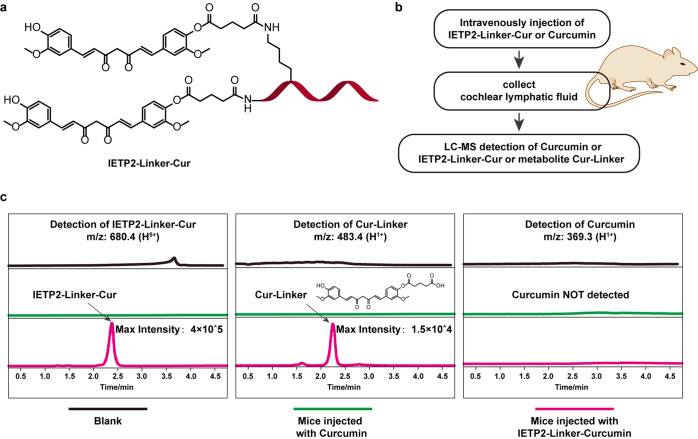
IETP2 delivers the small-molecule curcumin (Cur) across BLB. **a** Schematic representation of the structure of IETP2-Linker-Cur. Two Cur molecules were attached to one peptide molecule. The red ribbon represents IETP2. **b** Schematic representation of the processes for determination of the permeability of IETP2-Linker-Cur or Curcumin in mouse cochleae via intravenous administration. **c** MS traces showing the detection of IETP2-Linker-Cur, Cur-Linker or Cur from collected cochlear lymphatic fluid. To simplify the MS traces, specific cation peaks (*m*/*z*: 680.4 for IETP2-Linker-Cur, *m*/*z*: 483.4 for Cur-Linker, *m*/*z*: 369.3 for Curcumin) were detected for each compound. IETP2-Linker-Cur and Cur-Linker were detected in the cochlear lymphatic fluid from mice injected with IETP2-Linker-Cur. Curcumin was not detected in the cochlear lymphatic fluid from any of the mice

To validate whether the LRP1 pathway is a universal delivery system to cross the BLB, IETP2 was further conjugated with different compounds and tested for the ability to cross BLB. First, the natural product curcumin (Cur),^24,25^ which has been reported to protect against noise-induced hearing impairment,^26^ was conjugated to IETP2 (Fig. 4a and Supplementary Fig. S9a, b). The conjugate IETP2-Linker-Cur or Cur was intravenously injected into mice. Afterward, the mice were sacrificed, and cochlear lymphatic fluid was collected and subjected to liquid chromatography–mass spectrometry (LC–MS) for the detection of IETP2-Linker-Cur or Cur. Cochlear lymphatic fluid from untreated mice was used as a control (Fig. 4b). Cations corresponding to IETP2-Linker-Cur or Cur were displayed, showing that IETP2-Linker-Cur crossed the BLB and accumulated in the cochlear lymphatic fluid, while Cur did not effectively cross the BLB, and no detectable trace was found in the cochlear lymphatic fluid (Fig. 4c). To rule out the possibility of a poor signal response of Cur, collected blank cochlear lymphatic fluid was also mixed with Cur in vitro and subjected to LC–MS (Supplementary Fig. S9c), and the results showed a good signal response. Notably, in cochlear lymphatic fluid collected from mice treated with IETP2-Linker-Cur, the metabolite Cur-Linker was also detected, with relatively low intensity (Fig. 4c, middle panel). These results suggested that IETP2 can deliver Cur across the BLB and transport it to the cochlea.

Cur has been reported to be a potential drug for the treatment of noise-induced hearing impairment, and our data showed that Cur conjugated to IETP2 may be more likely to be effective. In a preliminary exploration, we evaluated the ototoxicity of IETP2, which showed no obvious ototoxicity at 7- and 14-days post-administration (Supplementary Fig. S10), providing further evidence that IETP2 is a safe therapeutic.

**Fig. 5:**
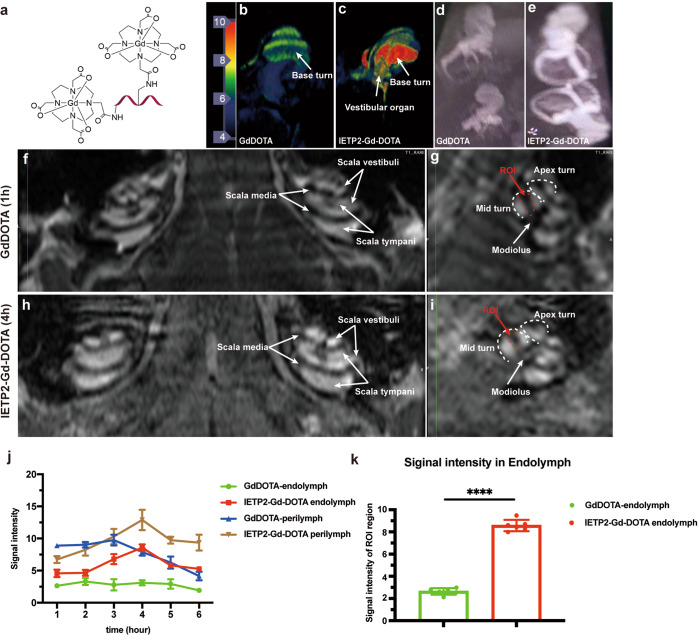
IETP2 delivers MRI contrast agent across the BLB. **a** Schematic representation of the structure of IETP2-Gd-DOTA. Two GdDOTA molecules were attached to one peptide molecule. The red ribbon represents IETP2. **b**, **c** 7.0 T MRI images of isolated cochleae from mice injected with IETP2-Gd-DOTA or free Gd-DOTA at 5 min after injection. Dosage: 1.5 mmol gadolinium/kg. The color bar on the left shows the MRI signal intensity; red indicates high signal intensity, while blue indicates low signal intensity. **d**, **e** 7.0 T MRI images of isolated cochleae of mice injected with IETP2-Gd-DOTA or free GdDOTA at 2 h after injection. Dosage: 1.5 mmol gadolinium/kg. **f**, **g** In vivo cochlear MRI images (**f** coronal plane; **g** para-sagittal plane) of mice injected with free GdDOTA at 1 h after intravenous injection. Dosage: 1.5 mmol gadolinium/kg. **f** The scala tympani and scala vestibuli were clearly enhanced, while the scala media remained at low intensity without enhancement. **g** Cross modiolus plane image of cochleae. An apex turn and midturn were seen (white dotted region). The ROI was the scala media region (red dotted region). **h**, **i** In vivo cochlear MRI image (**h** coronal plane; **i** para-sagittal plane) of mice injected with IETP2-Gd-DOTA at 4 h after intravenous injection. Dosage: 1.5 mmol gadolinium/kg. **h** Scala tympani and scala vestibuli were clearly enhanced, and scala media were also enhanced. **i** Cross modiolus plane image of cochleae. An apex turn and a midturn were observed (white dotted region). The ROI was the scala media region (red dotted region). Images captured at 1 or 4 h are displayed in **f**–**i** due to the saturated signal intensity at these time points. **j** Time-course signal intensity of the endolymph and perilymph in mice injected with IETP2-Gd-DOTA or GdDOTA. **k** Quantitative representation of the MRI signal intensity of the perilymphatic regions in (**g** and **i**) 4 h after drug injection. Error bars represent the mean ± SD. *N* = 6. ****p < 0.0001

Next, we investigated whether IETP2 can transport the magnetic resonance imaging (MRI) contrast agent gadolinium (Gd) across the BLB. Therefore, IETP2 was conjugated with GdDOTA (Fig. 5a and Supplementary Fig. S11). The conjugate IETP2-Gd-DOTA or free GdDOTA was injected into mice intravenously, and the ability to cross the BLB was directly validated by MRI. Cochleae were first harvested at different time points and subjected to magnetic resonance imaging. Five minutes post-injection, a very low MRI signal was observed in the GdDOTA-injected cochlea (Fig. 5b), while a strong signal was recorded for the IETP2-Gd-DOTA-injected cochlea (Fig. 5c). The same trend of MRI signal intensity was also observed at the 2-h time point (Fig. 5d, e), indicating that IETP2-Gd-DOTA can reach the cochlea more efficiently.

GdDOTA was an important contrast agent in enhancing the visualization of the perilymphatic space (Fig. 5f) but could not penetrate the BLB of the scala media to reach the endolymphatic region (Fig. 5g, ROI: endolymphatic region). Therefore, time-course MRI images were evaluated to determine whether IETP2 could direct Gd into the endolymphatic region. IETP2-Gd-DOTA reached the cochlear lymphatic fluid of mice 5 min after intravenous injection, while a much lower signal intensity was detected in mice injected with free GdDOTA (Fig. 5b, c). At 2 h post-injection, the signal intensity of IETP2-Gd-DOTA remained stronger than that of free GdDOTA (Fig. 5d, e), indicating prolonged retention of IETP2-Gd-DOTA in the lymphatic fluid. Moreover, in the in vivo images of IETP2-Gd-DOTA, signals could be detected in the perilymphatic space (Fig. 5h), endolymphatic region (ROI) and modiolus region (Fig. 5i, Red and White arrow) compared to lower intensity in the same region for the free GdDOTA group (Fig. 5g).

Next, we quantified the time-course signal intensity of GdDOTA or IETP2-Gd-DOTA in the perilymphatic and endolymphatic regions (Fig. 5j). Both GdDOTA and IETP2-Gd-DOTA reached the perilymphatic region in a time-dependent manner. GdDOTA did not reach the endolymphatic region within the measured 6 h. IETP2-Gd-DOTA showed time-dependent accumulation in the endolymphatic region, with peak accumulation at approximately 4 (Fig. 5j). The signal intensity of the endolymphatic regions of GdDOTA- and IETP2-Gd-DOTA-treated mice at 4 h post-injection is further displayed in Fig. 5k, which shows an approximately 3.3-fold signal intensity increase in the IETP2-Gd-DOTA group. In summary, these data indicated that IETP2 could direct GdDOTA molecules across the BLB and into the endolymphatic space.

## Discussion

The systemic delivery of therapeutic drugs to the inner ears is restricted by the BLB. In this study, we integrated a variety of tissue-specific high-throughput sequencing data, such as results from the peripheral auditory system related to the BLB, i.e., capillaries in the stria vascularis and modiolus, as well as the spiral vessel below the tunnel of Corti and to the sensory hair cells. In the central auditory system, data were obtained from the BBB, i.e., cerebral vascular endothelial cells, and from CNS neurons. The results were used to discover candidate targets to facilitate the passage of therapeutics across the BLB and the BBB. Using bioinformatics methods, a total of 1500 membrane-related genes were annotated, and 7 transmembrane protein genes were further selected as candidate targets. Based on previous knowledge, *Lrp1* was selected as the primary target. LRP1 protein is widely expressed in both the mouse and pig cochlea, especially in the regions of the BLB and target cells. Since LRP1 was reported to mediate the transcytosis of its ligands, we performed an in vivo biopanning assay to discover a peptide ligand for LRP1, aiming to establish a universal platform to deliver drugs across the BLB and into the inner ears.

A ligand peptide, IETP2, was thus discovered. IETP2 crosses the BLB and accumulates in both mouse and pig cochleae and targeting cells. We speculated that IETP2 may reach target cells through several pathways. First, IETP2 may be transported to the tunnel of Corti via spiral vessel endothelial cells and then to the IHCs and OHCs via inner and outer pillar cells and Deiters’ cells. Second, IETP2 may be transported to the SGN body via vascular endothelial cells within the modiolus and then spread to nerve fibers. Third, IETP2 may be transported to cells of the SV through the vascular endothelial cells of the SV. Fourth, IETP2 in the perilymph may be transported to the endolymph through the BM and vestibular or Reissner’s membrane.

By performing in vitro and in vivo experiments, we showed that IETP2 was partially colocalized with LRP1 in vivo and that the endocytosis of the peptide by cells was dependent on the expression of LRP1, suggesting the critical roles of LRP1 in transporting substances into inner ears. Considering the severe situation of inner ear disorders worldwide, the discovery of the transporting receptor LRP1 may play crucial roles in treating inner ear disorders in the future. Currently, the presence of the BLB impedes the systemic drug administration of drugs to the inner ear, while the discovery of LRP1 and its ligand IETP2 may provide a new avenue for noninvasive and systemic administration that is feasible for patients with inner ear disorders. We also validated the efficient delivery of the MRI contrast agent gadolinium and the potential therapeutic curcumin using the binding peptide IETP2. These studies lay the groundwork for future improvements in the noninvasive diagnosis and treatment of inner ear disorders. For example, strategies for conjugating various drugs to IETP2 should be explored for transporting molecules across the BLB and directing therapeutics into target cells within the cochlea.

Strategies of conjugating the therapeutic drugs to our discovered IETP2 peptide should be further and more broadly explored to evaluate the efficacy of the strategies in protecting hearing from noise and in restoring hearing in cases of sudden hearing loss and Meniere disease. Moreover, inner ear diseases are not limited to the peripheral auditory system, and the ascending auditory pathway and central auditory system are also involved in IEDs, such as presbycusis. Since LRP1 is also widely expressed in neurons in the brain and BBB, the LRP1 pathway might illuminate the treatment of IEDs both with and without central auditory system changes. We believe that a number of novel drugs or nanomaterial drug-delivery systems could be developed based on the LRP1 pathway. In addition, we found 6 other candidate membrane receptors, which remained unexplored in our manuscript. Further exploration of the 6 candidates may further enrich the solutions proposed for crossing the BLB.

## Materials and methods

### Animals and ethics approval

All animal experiments performed in this study met the National Natural Science Foundation of China (NSFC) guidelines for the care and use of laboratory animals and were approved by the Animal Ethics Committee of the People’s Liberation Army (PLA) General Hospital. Wild-type C57BL/6J and BALB/c nude mice provided by Vital River Laboratories (Beijing, China) were raised and bred in the Animal Center of the PLA General Hospital under specific pathogen-free (SPF) conditions. Pigs at the age of 3 to 5 days postnatal were provided by Beijing Strong Century Minipigs Breeding Base (Beijing, China), and pigs were raised under conventional conditions. Pigs were sacrificed two hours after injection on the day of delivery. We did not house and maintain pigs in the animal center.

### Bioinformatics analysis

The terms mouse “Vascular endothelial cell related to blood–brain barrier (BBB)” (GSM4222733, GSM4222734, GSM4222733), “CNS neuron” (GSM4125956, GSM4125957, GSM4125958, GSM4125959) and “hair cell (HC)” (SRR1616830, SRR1616831, SRR1616832) were searched and downloaded from the Gene Expression Omnibus (GEO) database (https://www.ncbi.nlm.nih.gov/geo/). The SGN and SV data were acquired by RNA-seq using modiolus or SV tissue dissected from mouse cochleae in our laboratory. Then, the above five groups of raw data were processed into transcriptome expression data and standardized to the TPM format for Gene Ontology (GO) annotation. A total of more than 20,000 genes and a total of 5403 cell membrane function-related genes were annotated, and we removed genes with inconsistent expression within the same group (FilterExpression). Finally, approximately 1500 overlapping genes were selected for further analysis (heat-map and GO enrichment analysis) as described in a previous study.^27^

### Construction of a phage-displayed random peptide library and mouse-based, phage-display-assisted in vivo panning

A phage-displayed random peptide library was first constructed based on the backbone of ANG2. For in vivo panning, the phage-displayed library was amplified in the *E. coli* ER2738 strain, and 10^11^ plaque-forming units (pfu) of phages in 100 μL of PBS were intravenously injected into C57BL/6J mice. Two hours later, the mice were sacrificed. After perfusion, the cochleae were collected and homogenized. The supernatant of the homogenate was used to infect the ER2738 strain, and the sustained phages were further amplified in the ER2738 strain and used for the next round of in vivo panning. A total of four rounds of in vivo panning were performed. Finally, four of the most enriched clones were sequenced with the M13 primer (5’-HOCCC TCA TAG TTA GCG TAA CG-3’), and the corresponding peptides were used and validated in the following experiments.

### Western blot and quantitative real-time polymerase chain reaction (qPCR)

Cochlear proteins were extracted from six C57BL/6J mice (either sex, 4 weeks old) and one Bama miniature pig (male, P5). All tools were placed on ice before the operation, and cochlear protein was extracted via lysis with RIPA lysis buffer. Western blotting was performed as previously described. Proteins were separated in a 10% sodium dodecyl sulfate-polyacrylamide gel and then transferred onto a polyvinylidene difluoride membrane (Millipore, Bedford, MA). The PVDF membrane was blocked using 8% nonfat milk (CST, 9999 s) at RT for 2 h and then immunoreacted with anti-LRP1 (Invitrogen MA5-31959, 1:1000) and anti-GAPDH (CST, 5174S, 1:3000) antibodies at 4 °C overnight. Both antibodies were diluted using 5% nonfat milk diluted with TBST (1× working solution). The protein bands were visualized with an ECL Plus (CST, 12630P) chemiluminescence detection system (GE Healthcare). The exposure time period was 30 s, and the signal intensity was analyzed using ImageJ and Prism 8.0.

Cochlear total RNA was extracted from the six C57BL/6J mice (either sex, 4 weeks old) and one Bama miniature pig (male, P5) by using TRIzol reagent (Tiangen) according to the manufacturer’s protocol, followed by denaturation at 95 °C for 30 s and annealing and elongation temperature at 60 °C for 2 min, for 25 cycles. Complementary DNA was synthesized from 1 μg of total RNA by using an ImProm-II Reverse Transcription System (Promega, USA). To assess gene amplification, qPCR was performed by using SYBR Green. The expression of β-actin, the internal standard, was arbitrarily assigned a value of 1.0. The sequence-specific primers for Lrp1 and LRP1 qPCR were as follows: F: TCCTGGCTACGTACCTGAGT and R: ATGGTGTGCTCATCCACGAA; GAPDH, F: ACTCACTCTTCTACCTTTGATGCT and R: TGTTGCTGTAGCCAAATTCA.

### In vivo and ex vivo fluorescence imaging

BALB/c nude mice (4 weeks old, either sex) were divided into three groups (5 mice for each group). Each group was injected with saline, Gly-Cy5.5 or IETP2-Cy5.5 via the tail vein. After the indicated times, the animals were anesthetized with isoflurane and subjected to live animal imaging (IVIS Lumina III, Caliper LifeSciences, MA, USA). The excitation/emission filter was set at 625/700 nm, and images were taken with an interval of 20 min post-injection. To validate the uptake efficiency of different IETPs, mice were sacrificed 1 h post-injection, and the cochleae were collected and imaged. To evaluate the specificity of IETP2 in targeting the cochleae, brain and muscle were also collected and imaged. The time-dependent uptake of IETP2 in mouse cochleae was assessed at a series of time points (1, 1.5, 2, and 2.5 h) with 3 mice at each time point for each group. Collected images were analyzed using Living Image software (IVIS Imaging System, 4.5.5).

### Immunostaining and histological analysis

Four-week-old C57BL/6J mice of either sex were harvested 50 min and 2 h post-injection. Cochleae were fixed in 4% paraformaldehyde at 4 °C for 8 h and decalcified in 4% EDTA for 4 days at room temperature (RT). Parts of the cochleae were dissected into pieces containing the basilar membrane (BM), organ of Corti (OC) and stria vascularis (SV) from the apex to the hook region and then used for whole-mount immunofluorescence. Other parts of the cochleae were dehydrated using a graded sucrose solution (15% and 30%; 3 h for each grade) at RT, embedded in optimum cutting temperature (OCT) compound for 5 h at RT, frozen for 1 h at −20 °C and sectioned with a freezing microtome (Leica CM1900) at an 8 μm thickness.

For whole-mount and cross-section immunofluorescence, tissues were incubated with 0.1% Triton X-100 and blocked with 10% donkey serum for 2 h at RT before the application of the primary antibodies. The tissues were incubated with a rabbit anti-LRP1 monoclonal antibody (1:100, Invitrogen MA5-31959) and a mouse anti-ZO1-647 antibody (1:200, Invitrogen MA3-39100-A647) in PBS containing 1% Triton X-100 at RT overnight. The tissues were then incubated with the following secondary antibodies for 1 h after three rinses with PBS at RT on a shaker: Alexa Fluor 488-conjugated donkey-anti-rabbit (A32790), Alexa Fluor 555-conjugated donkey-anti-mouse (A-31570) and Alexa Fluor 555-conjugated phalloidin (A34055) (all from Invitrogen; all at a 1:1000 dilution). After three rinses with PBS, the specimens were mounted with mounting solution containing DAPI (Invitrogen, P36931). Confocal images were obtained with a Zeiss LSF980 microscope using a 10×, 20× or 63× glycerin-immersion lens with or without digital zoom. Lenses of 10× and 20× were used to obtain an overall view of the tissue, and lenses of 63× were used to observe the region of interest. For whole-mount tissues, snap and z-stack images of apex, middle and base turns were obtained to observe OHCs, IHCs and different layers of the OC. ImageJ (NIH Image) was used to analyze the confocal images and to acquire the maximum-intensity projections of the z-stacks of each segment.

### Synthesis and use of Lix-FITC

Lix-FITC, a polypeptide consisting of an ANG2 sequence and labeled with fluorescein isothiocyanate (FITC), was synthesized by Nanjing Peptide Industry Co., Ltd. The sequence of this peptide was FITC-Acp–His–Gly–Glu–Gly–Thr–Phe–Thr–Ser–Asp–Leu–Ser–Lys–Gln–Met–Glu–Glu–Glu–Ala–Val–Arg–Leu–Phe–Ile–Glu–Trp–Leu–Lys–Asn–Gly–Gly–Pro–Ser–Ser–Gly–Ala–Pro–Pro–Ser–Lys–Lys–Lys–Lys–Lys–Lys–Gly-Gly-Ser-ANG2. Lix-FITC was first used as a primary antibody to locate LRP1 in the cochleae and also as a competitor ligand of IETP2 to test whether the IETP2-mediated delivery of small molecules was mediated by the LRP1 receptor.

### In vitro experiments using the HEI-OC1 cell line

The House Ear Institute-Organ of Corti 1 (HEI-OC1) cell line was derived from the auditory organ of a transgenic mouse and is one of the few auditory cell lines that can be used for research purposes.^28,29^ The HEI-OC1 cell line is usually cultured under permissive conditions (33 °C, 10% CO_2_), which only accelerates its proliferation. Under nonpermissive conditions (39 °C, 5% CO_2_), differentiation is triggered. In this manuscript, HEI-OC1 cells (WT or LRP1-knockdown) were incubated in Dulbecco’s modified Eagle’s medium (DMEM) (Gibco, 22320030) supplemented with 10% fetal bovine serum (Gibco, FBS) under permissive conditions. For the Lix-FITC competing assay, WT HEI-OC1 cells were first seeded in confocal microscopy dishes and cultured for 24 h. The cells were then incubated with the indicated amount of IETP2 peptide and/or Lix-FITC for 45 min. For competition, Lix-FITC was preincubated with cells for 10 min before the addition of IETP2. After incubation, the cells were maintained in a live-cell imaging container, and a Zeiss LSF980 microscope was used to record the images. In the LRP1-knockdown experiments, WT and LRP1-knockdown cells were incubated with IETP2 for 45 min and subjected to microscopy imaging or flow cytometry. Microscopy images were analyzed with Zen or ImageJ software, and figures were arranged with Prism 8.0 (GraphPad). Flow cytometry data were analyzed with FlowJo.

### LC–MS-based detection of IETP2-Linker-Cur in cochlear lymphatic fluid

C57BL/6 J mice injected with IETP2-Linker-Cur intravenously were sacrificed 2 h after injection, cochleae were harvested with intact round windows and oval window membranes, and cochleae with damaged RWM and OWM were used for other experiments, such as immunostaining. After rinsing in PBS (1×) three times (30 s each time), the isolated cochleae were dried with a small cotton swab and placed on a glass cell culture plate (Corning). A small hole was made with forceps on the apex turn of each cochlea. A homemade manual microsyringe pump composed of five parts was used to withdraw the inner ear lymphatic fluid (a mixture of perilymph and endolymph). As shown in Fig. S12, the homemade manual microsyringe pump was made of five parts: **a** was the micrometer to control the volume (the injection volume range was 1–10 µL), **b** was the microinjector (HAMILTON 80001), **c** was the microtip connecting b and d (WPI, 501656), **d** was the FEP Teflon microdialysis tubing (WPI, 505515), and **e** was the polyimide tubing (A-M Systems, 823400) connected to Teflon tubing. To prevent possible contamination from cerebrospinal fluid, we withdrew a maximum of 1 µL per cochlea. For each group, a total of 20 mice were sacrificed, and 20 µL of lymphatic fluid was extracted for testing. All tools involved were autoclaved before the experiment.

The inner ear lymphatic fluid (approximately 20 μL) was collected, and 80 μL of pure acetonitrile (Merck Company, Germany) was added. The mixture was centrifuged at a speed of 20,000 × *g* for 20 min at 24 °C, and the supernatant was collected. A 2 μL sample was subjected to LC–MS equipped with a Capcell Pak C8 column (3 μm, 50 × 2.0 mm, Shiseido, Japan). Water and acetonitrile supplemented with 0.1% formic acid served as mobile phases A and B, respectively. The flow rate was set to 0.7 mL/min with the following linear gradient LC method: 50% B at 0 min, 60% B at 1.5 min, 80% B at 2.5 min, 98% B at 3.5 min, 98% B at 4.5 min, and 50% B from 4.6 min to 6 min. To simplify the MS traces, specific cation peaks (*m*/*z*: 680.4 for IETP2-Linker-Cur, *m*/*z*: 483.4 for Cur-Linker, *m*/*z*: 369.3 for Cur) were detected for each compound.

### Animal MRI

MRI was performed with a 7.0 T scanner (USR Biospec Bruker 70–20, Germany). Since the scanning diameter was 30 cm, which was appropriate for small animals, C57BL/6J mice (6 weeks old, weight 20–25 g, either sex) were used in these sets of experiments.

Mice were anesthetized with 1% pentobarbital sodium (100 μL/10 g, intraperitoneal [i.p.]). The body temperature of each animal was maintained during testing with a circulating heating pad (37 °C), and 0.5–1.5% isoflurane was used to maintain anesthesia inside the magnet. The respiration rate was monitored during scanning. Then, GdDOTA (0.5 mmol/mL) was diluted to 100 μL with saline and injected via the tail vein (1.5 mmol/kg), and IETP2-GdDOTA was dissolved into 100 μL of saline and administered via *i.p*. injection (0.75 mmol/kg; each peptide was conjugated with two GdDOTA molecules). Therefore, the amount of Gd injected into each animal of identical weight was equal.

T1-weighted 3-dimensional images were acquired by using the following parameters: a gradient echo 3D sequence, a repetition time (TR) of 300 ms, an echo time (ET) of 12.66 ms, a number of averages of 20, a field of view of 10×4.2×5 mm^3^, a slice thickness of 0.1 mm, a rare factor of 4, a size of 200×84×50 pixels and a resolution of 0.05 × 0.05 × 0.1 mm^3^. ImageJ (NIH) was used to carry out postproduction processing of images, including the measurement and adjustment of the signal intensity in the endolymph; the quantification of the signal intensity at ROIs; and labeling and demonstrating the presence of perilymph in the scala tympani, scala vestibuli and vestibulum and of endolymph in the scala media and vestibulum. The T1-weighed GdDOTA and IETP2-Gd-DOTA relaxation rates of the samples were analyzed by Prism 8.0 (GraphPad) (Supplementary Fig. S5).

### In vitro LRP1 knockdown by lentiviral delivery of CRISPR/Cas9 in the HEI-OC1 cell line

To efficiently knock down the LRP1 gene, we designed different sgRNAs using MIT CRISPR Design and Broad Institute CRISPRko targeting exon 2 and exon 3. We finally selected two sgRNAs with high on-target scores and optimal off-target scores. The sequence of sgRNA1 was ATTGTGTACCTACACCCAGT; that of sgRNA2 was ACCGTATGCTGGGTGCACGT. The sequences of the plasmid for lentivirus (LV) construction, pLV-lenti.EFS.hspCas9.U6.sgRNA, were designed by PackGene Biotech (Guangzhou, China), and the LVs were packaged by the same company (Supplementary Fig. S8). HEI-OC1 cells were incubated in 6-well plates at 37 °C with 10% CO_2_ in DMEM (Gibco, 22320030) supplemented with 10% fetal calf serum (Gibco, FBS). One day later, the cells were transduced with 50 µL of LV-lenti.EFS.hspCas9.U6.sgRNA1 and LV-lenti.EFS.hspCas9.U6.sgRNA2 (2.58 × 10^8^ TU/mL). One day post-transduction, the medium was replaced with fresh DMEM. Three days post-transduction, puromycin dihydrochloride (Beyotime ST551) was added to DMEM with 10% FBS at 6 mg/mL to select for the cells infected by LV. After three rounds of selection, every 3 days, the cells were seeded into 24-well plates for in vitro experiments.

### Acoustic testing

Auditory brainstem response (ABR) testing was conducted in a standard soundproof chamber for animal use. Mice were anesthetized with a mixture of isoflurane (Keyue Life Science Co., Ltd.) and air via a respirator for animal use (Medical Supplies and Services Int., Ltd.) and an aquarium air pump (HAILEA, A00–5505). The final concentration of isoflurane was 2% at a flow rate of 1.5. Circulating heating pads were placed under the mice to maintain body temperature at 37 °C. ABRs were recorded with a Tucker Davis Technologies (TDT) RZ6 instrument, and the sound was delivered by a calibrated loudspeaker (TDT, MF1 2356).

For ABR testing, needle electrodes were inserted beneath the skin. The recording electrode was inserted at the vertex, the reference electrode was inserted at the pinna, and the ground electrode was inserted near the tail. The ABR stimuli were frequency-specific tone pips at frequencies from 1 kHz to 32 kHz with a duration of 5 ms (0.5 ms rise-fall delivered at 21.1/sec), and each response was amplified 10,000-fold with a filter passband of 100 Hz to 3 kHz and averaged over 1024 times. The signal started at 90 dB sound pressure level (SPL) with a step of 10 dB and decreased to 10 dB SPL at frequencies of 4, 8, 16, 24, and 32 kHz for mice. The threshold was defined as the lowest dB SPL with an identifiable and repeatable wave I for mice. The results were interpreted by three independent otologists without knowledge of the subgroup of each animal, and the average hearing threshold of each group at each frequency was used for further data analysis.

### Statistical analyses

One-way ANOVA was used to compare the differences in ABR thresholds between groups, and Student’s unpaired t test was used to compare the signal intensity of fluorescence and the signal intensity of MRI between groups. Differences for which *p* < 0.05 were considered statistically significant. The analyses were conducted in Prism 8.0 (GraphPad). Adobe Illustrator 2020 was used to construct the figures.

## Supplementary information

Supplemental figures

## Data Availability

All data that support this research are available in this manuscript and its supplemental materials.
