# Supplementary material for: LDL receptor-related protein 1 (LRP1), a novel target for opening the blood-labyrinth barrier (BLB)
Source: Signal Transduct Target Ther. 2022 Jun 10;7:175. doi: 10.1038/s41392-022-00995-z (PMC9184653; doi:10.1038/s41392-022-00995-z)
Supplement: Supplementary file 1 — Supplemental figures [file 41392_2022_995_MOESM1_ESM.docx]

Supplementary Materials for

**LDL receptor-related protein 1 (LRP1), a novel target for opening the blood-labyrinth-barrier (BLB)**

Xi Shi, Zihao Wang, Wei Ren, Long Chen, Cong Xu, Menghua Li, Shiyong Fan, Yuru Xu, Mengbing Chen, Fanjun Zheng, Wenyuan Zhang, Xinbo Zhou, Yue Zhang, Shiwei Qiu, Liyuan Wu, Peng Zhou, Xinze Lv, Tianyu Cui, Yuehua Qiao, Hui Zhao, Weiwei Guo, Wei Chen, Song Li, Wu Zhong, Jian Lin, Shiming Yang

Correspondence to: [shm_yang@163.com](mailto:shm_yang@163.com), [Linjian@pku.edu.cn](mailto:Linjian@pku.edu.cn), [zhongwu@bmi.ac.cn](mailto:zhongwu@bmi.ac.cn),

**This PDF file includes:**

Figures. S1 to S12

Tables S1 to S2


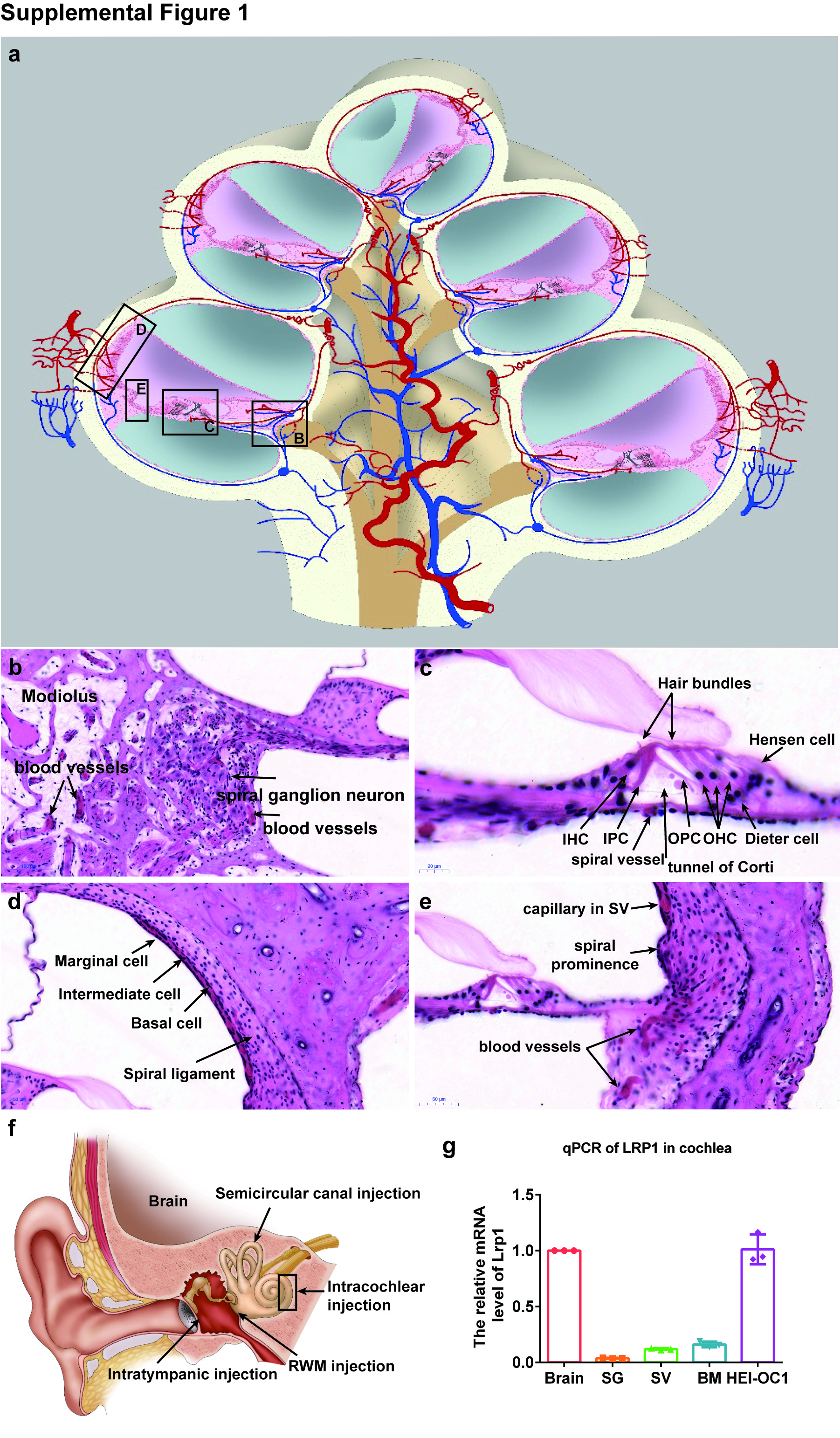


**Supplemental Figure 1 BLB distribution in the cochlea (related to Figure 1).**

**a**. Schematic overview of the cochleae at cross section plane and the blood vessel distribution in the cochleae. **b**. A zoomed-in CE-HE image of the modiolus and ganglion cell regions. Blood vessels could be seen in the modiolus and ganglion regions, and ganglion neurons could be seen. **c**. A zoomed-in CE-HE image of the basilar membrane and organ of Corti (OC). The microstructure of the OC was shown, which consisted of IHCs, OHCs, inner pillar cells (IPC) IPCs, outer pillar cells (OPCs), Dieter cells and Hensen cells. Hair bundles of HCs could also be seen. The tunnel of Corti was the space between the IPC, OPC and basilar membrane, which was filled with perilymph. The spiral vessel was located beneath the tunnel of Corti and was responsible for the nutritious supply of OC. **d**. A zoomed-in CE-HE image of stria vascularis. SV is the main contributor to the BLB in the cochleae and consists of three layers of cells, marginal cells, intermediate cells and basal cells. Capillaries could be seen inside the SV. **e**. The magnified CE-HE image of spiral prominence, which was the region between the OC and SV with capillaries inside. **f**. Schematic diagram of various methods of local administration of therapeutics into the inner ear, including intratympanic injection, round window membrane (RWM) injection, intracochlear injection and semicircular canal injection. **g**. The qPCR results of LRP1 in different tissues in mouse (brain, SG, SV and BM and OC) and HEI-OC1 cell lines. (Samples used in g were obtained from mice.)


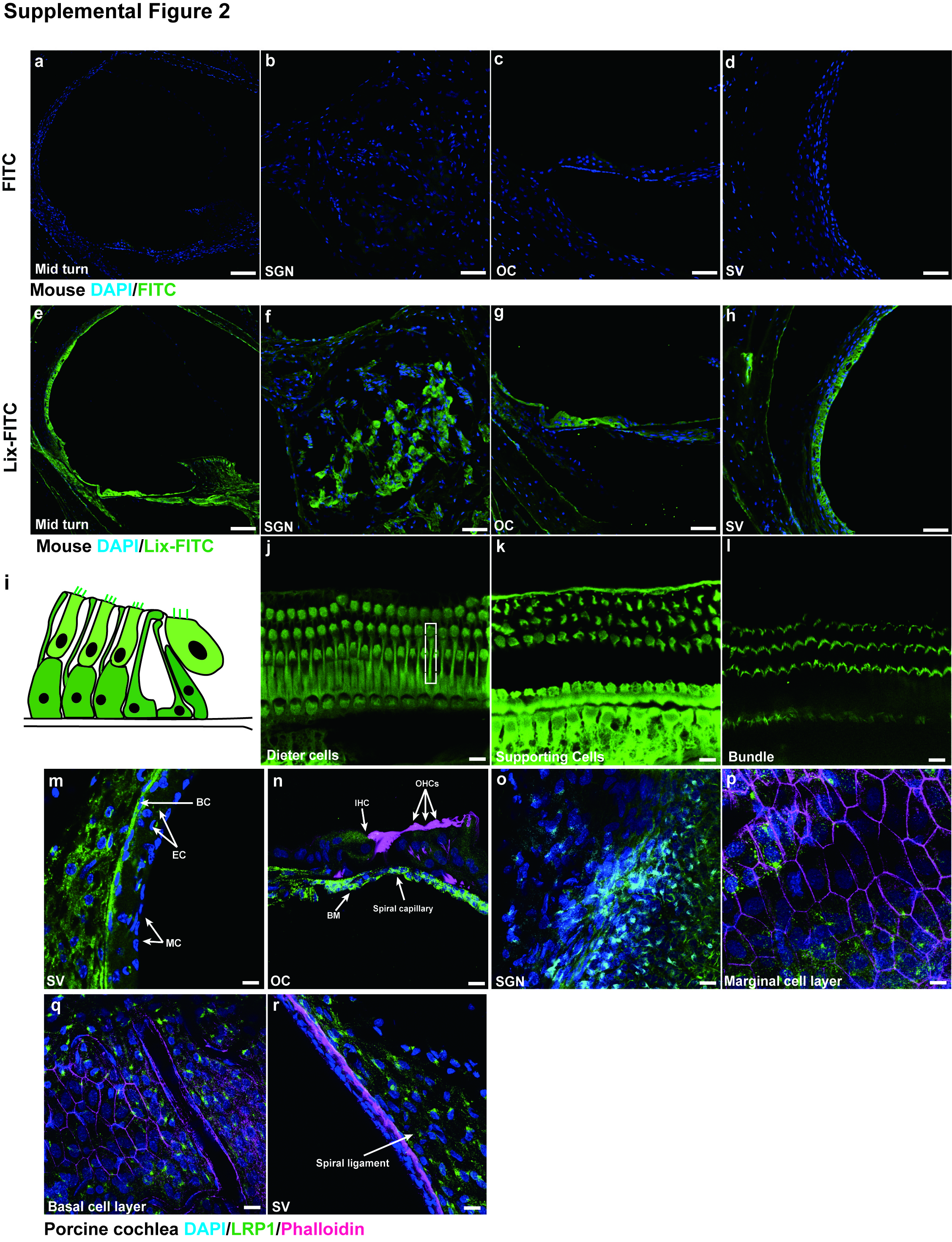


**Supplemental Figure 2 Localization of the receptor LRP1 in cochleae (related to Figure 1)**.

**a-d**. Immunofluorescence images of a WT mouse cochlea incubated with Alexa Fluor 488 (TRITC)-conjugated secondary antibodies without primary antibodies (cross section). **e-h**. Immunofluorescence images of a WT mouse cochlea incubated with the LRP1 receptor ligand Lix-FITC without primary antibodies (cross section). LRP1 localized in the ganglion neuron body, SV cells and BM. **i**. Schematic overview of the organ of Corti. LRP1 (green) was localized in HCs, hair bundles and supporting cells. **j-i**. Whole-mount cochlear tissue incubated with Lix-FITC alone. LRP1 localized to Dieter cells and hair bundles. **m-r**. Immunofluorescence images of LRP1 by anti-LRP1 antibody in pig cochlea. LRP1 receptor was located in basal cells in SV, endothelial cells in SV and cells of BM. Phalloidin was used to locate endothelial cells, marginal cells and HCs. (DAPI: blue; LRP1: green; phalloidin: magenta. Scale bars for a and e represent 100 µm, m, n, p and q represent 5 µm, and the others represent 20 µm.


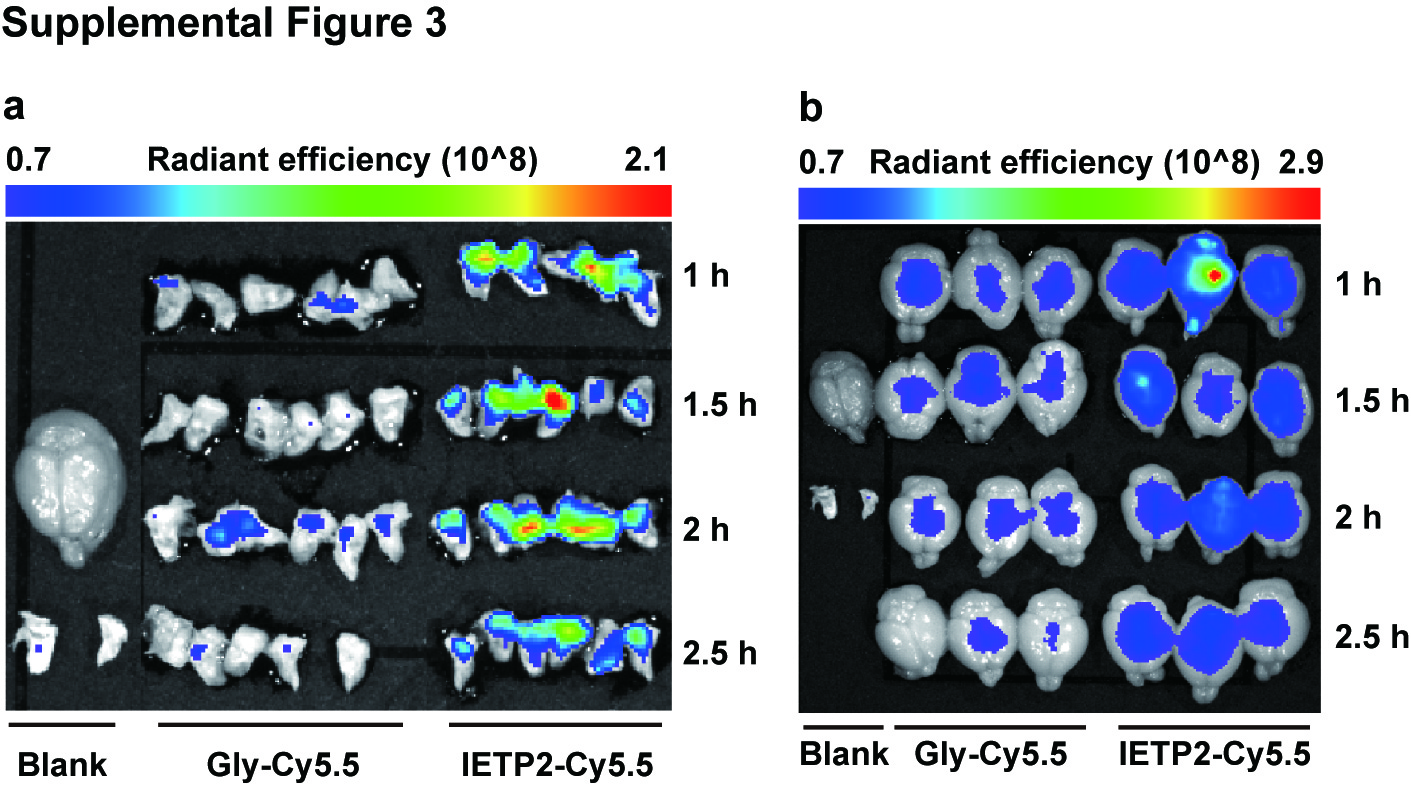


**Supplemental Figure 3. *Ex vivo* imaging showing the permeability of IETP2 in the brain and inner ear (related to Figure 2).**

**a.** *Ex vivo* imaging showing the time-lapse accumulation of IETP2-Cy5.5 in mouse cochleae. Gly-Cy5.5 served as a negative control. **b.** *Ex vivo* imaging showing the time-lapse accumulation of IETP2-Cy5.5 in mouse brains. Gly-Cy5.5 served as a negative control.


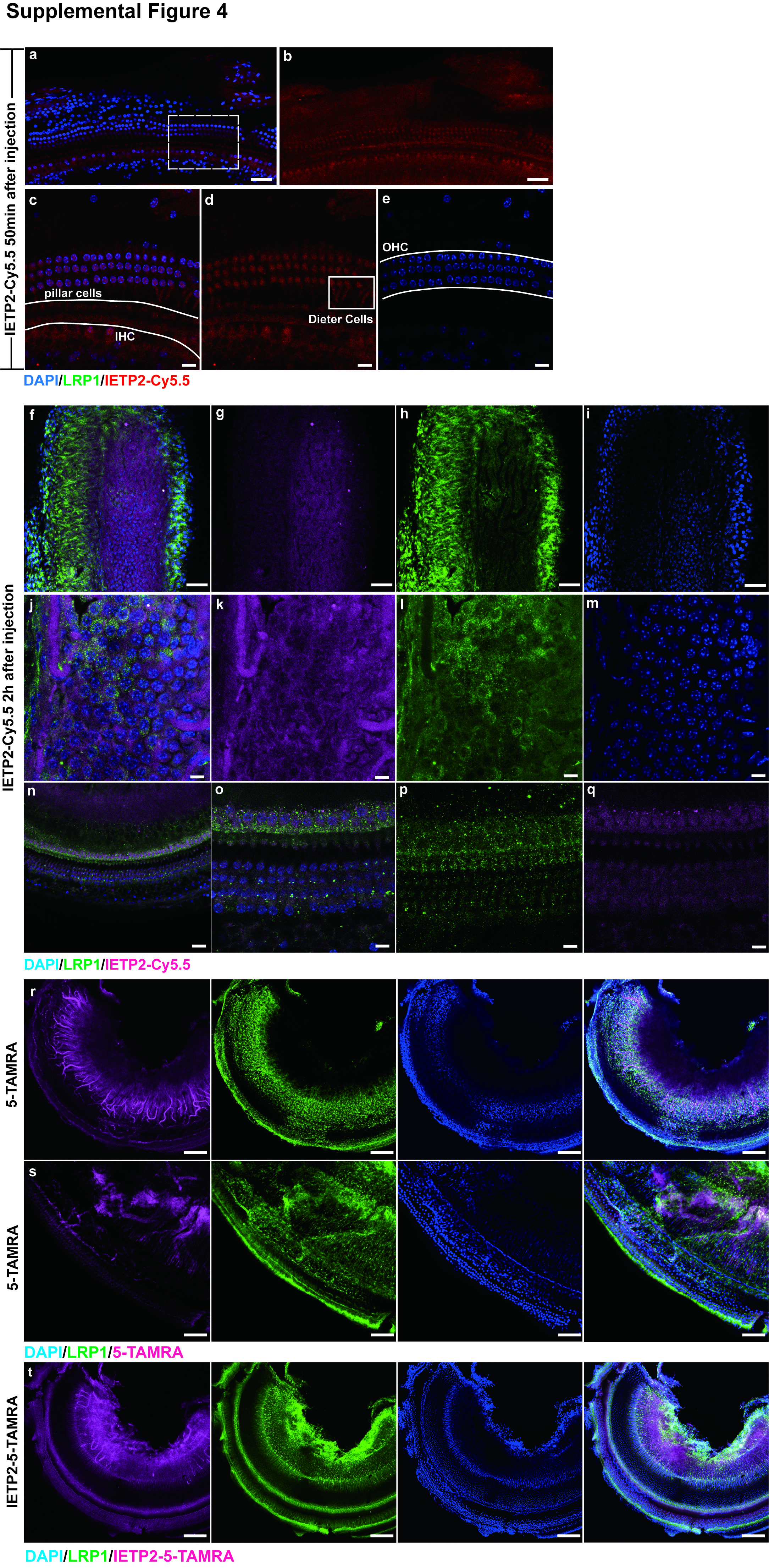


**Supplemental Figure 4.** Whole-mount images of mouse cochleae **50 min and 2 h after tail vein injection of IETP2-Cy5.5 (related to Figure 2)**.

**a-e**. Images 50 min after intravenous injection of IETP2-Cy5.5. a and b Overview of the OC at lower magnification with or without a DAPI signal. c-e are magnified regions of the images in A1 (white dotted rectangle). The IETP2-Cy5.5 signal could be detected in IHCs, OHCs, pillar cells and Dieter cells (white rectangle). **f-q**. Cochlear whole-mount tissue of mice injected with IETP2-Cy5.5 at 2 h after injection was incubated with anti-LRP1 antibody (green). f-i Overview of the SV at a relatively low magnification of 20×. j-m show the marginal cell layer of the SV. IETP2-Cy5.5 was transported into marginal cells. n-q shows that IETP2-Cy5.5 was transported into HCs, especially IHCs. **r** shows the immunofluorescence images of the BM layer of mouse cochleae after intravenous injection of free 5-TAMRA. **s** Immunofluorescence images of the OC layer of mouse cochleae after intravenous injection of free 5-TAMRA. **t** shows the immunofluorescence images of the OC layer of mouse cochleae after intravenous injection of IETP2-5-TAMRA. The images in C1-3 are Z-stack images of 9 layers. The tissues were stained with anti-LRP1 antibody and AF488 secondary antibody, and a lower LRP1 signal was detected in the IETP2-5-TAMRA group than in the free 5-TAMRA group in C2. (DAPI: blue; LRP1: green; phalloidin: magenta. Scale bars are 200 µm for r and t, 100 µm for s, 50 µm for a, b and f-j, and 10 µm for c, d, e and j-q.)


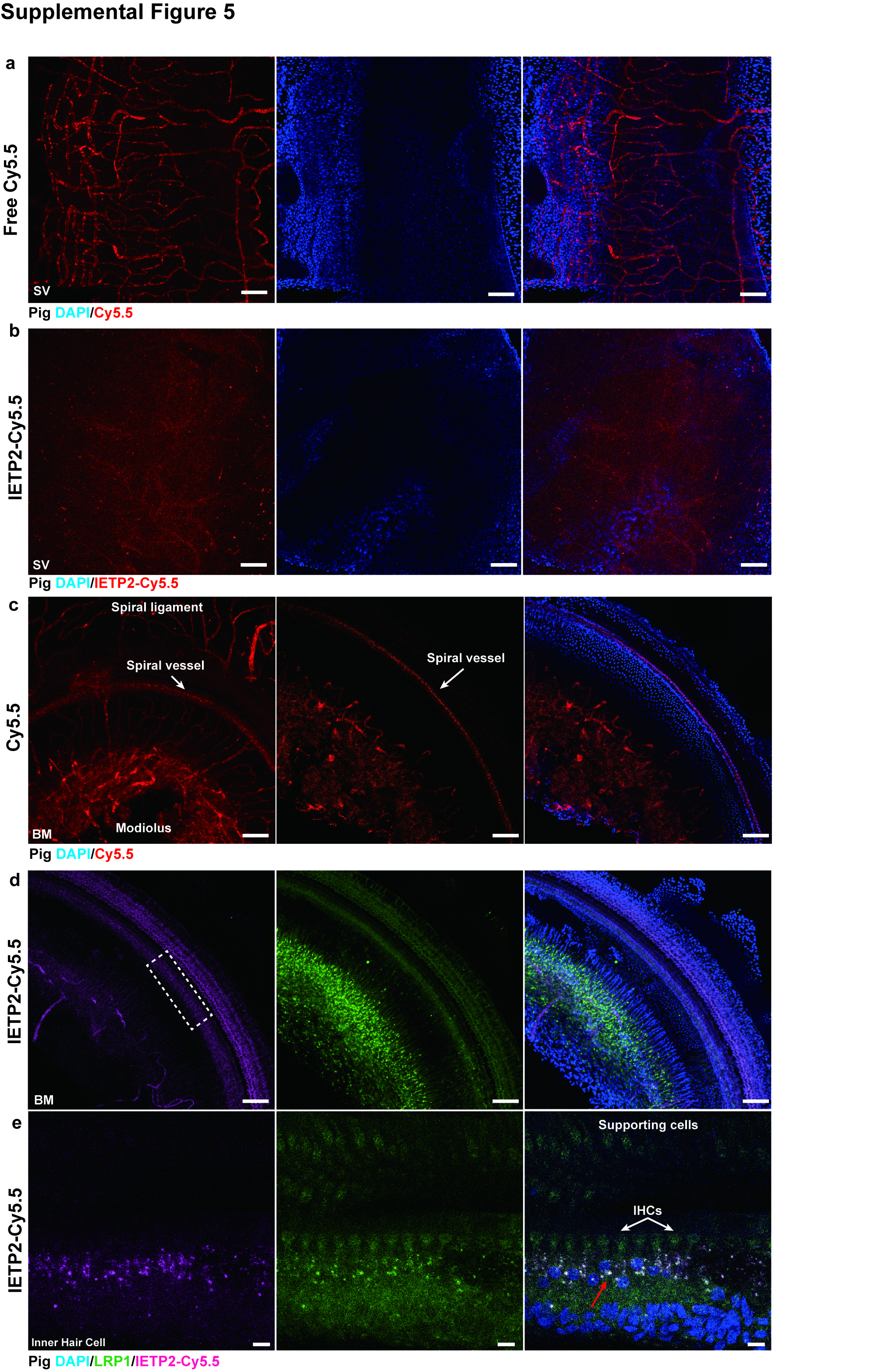


**Supplemental Figure 5** Pig **cochlear immunofluorescence images 2 h after intravenous injection of free Cy5.5 and IETP2-Cy5.5 (related to** Figure 2**)**.

**a.** SV images after injection of Cy5.5; signals were retained inside the blood vessels. **b**. SV images after injection of IETP2-Cy5.5; signals could be detected in cells outside the capillary wall. **c**. BM images after injection of Cy5.5, which showed the blood vessel distribution of the BM. Signals were restricted in blood vessels. Spiral capillaries are clearly visible (white arrow). **d**. BM and OC images after injection of IETP2-Cy5.5 and incubation with an anti-LRP1 antibody. Signals could be observed in IHCs and OHCs. **e**. The zoomed-in OC images in the white dotted rectangle in **f**. Signals in IHCs could be observed, and colocalization of LRP1 and IETP2-Cy5.5 could be seen (red arrow). (DAPI: blue; LRP1: green; IETP2-Cy5.5 or Cy5.5: red or magenta. Scale bars are 10 µm for e and 100 µm otherwise.)


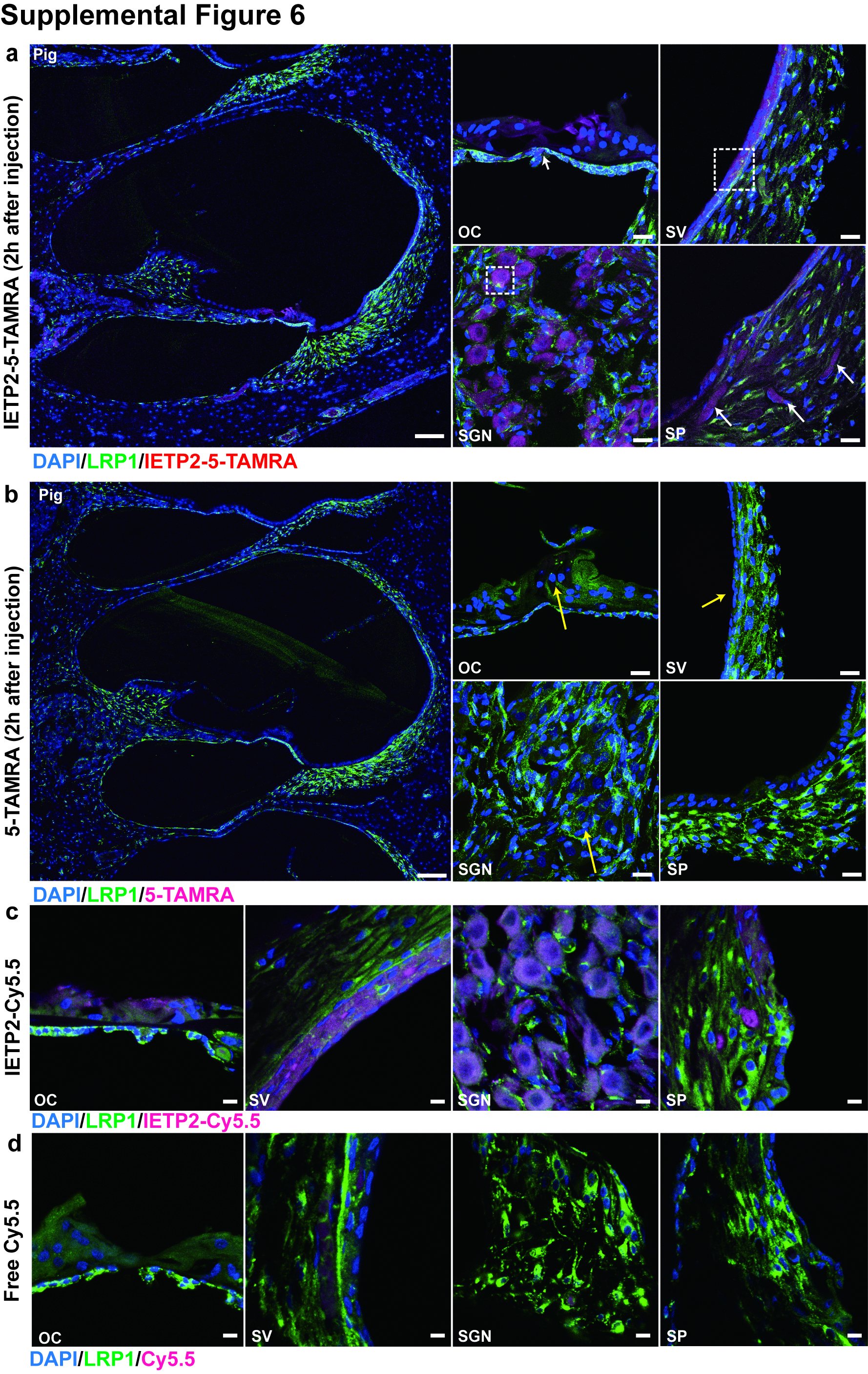


**Supplemental Figure 6 Pig cochlear immunofluorescence images 2 h after intravenous injection of 5-TAMRA, IETP2-5-TAMRA, Cy5.5 and IETP2-Cy5.5 (related to Figure 2).**

**a**. Images from the IETP2-5-TAMRA injection group. 5-TAMRA was transported into targeted areas, such as HCs, ganglion neurons and the SV, with some retention in the blood vessels (white arrows). Colocalization of IETP2-5-TAMRA and LRP1 was observed in ganglion neurons and the SV (white dotted rectangle). **b**. Images from the 5-TAMRA injection group. Free 5-TAMRA was not observed on the surfaces of IHCs, some OHCs, ganglion neurons or the SV (yellow arrow). The setting parameters for the microscope (including the excitation intensity and emission gain) remained consistent throughout the experiments. **c**. Images of pig cochleae injected with IETP2-Cy5.5 (2 h after injection). **d**. Images of pig cochleae injected with free Cy5.5 (2 h after injection). (DAPI: blue; LRP1: green; phalloidin: magenta. Scale bars for the overview of porcine cochlear mid turn are 200 µm; others are 10 µm.)


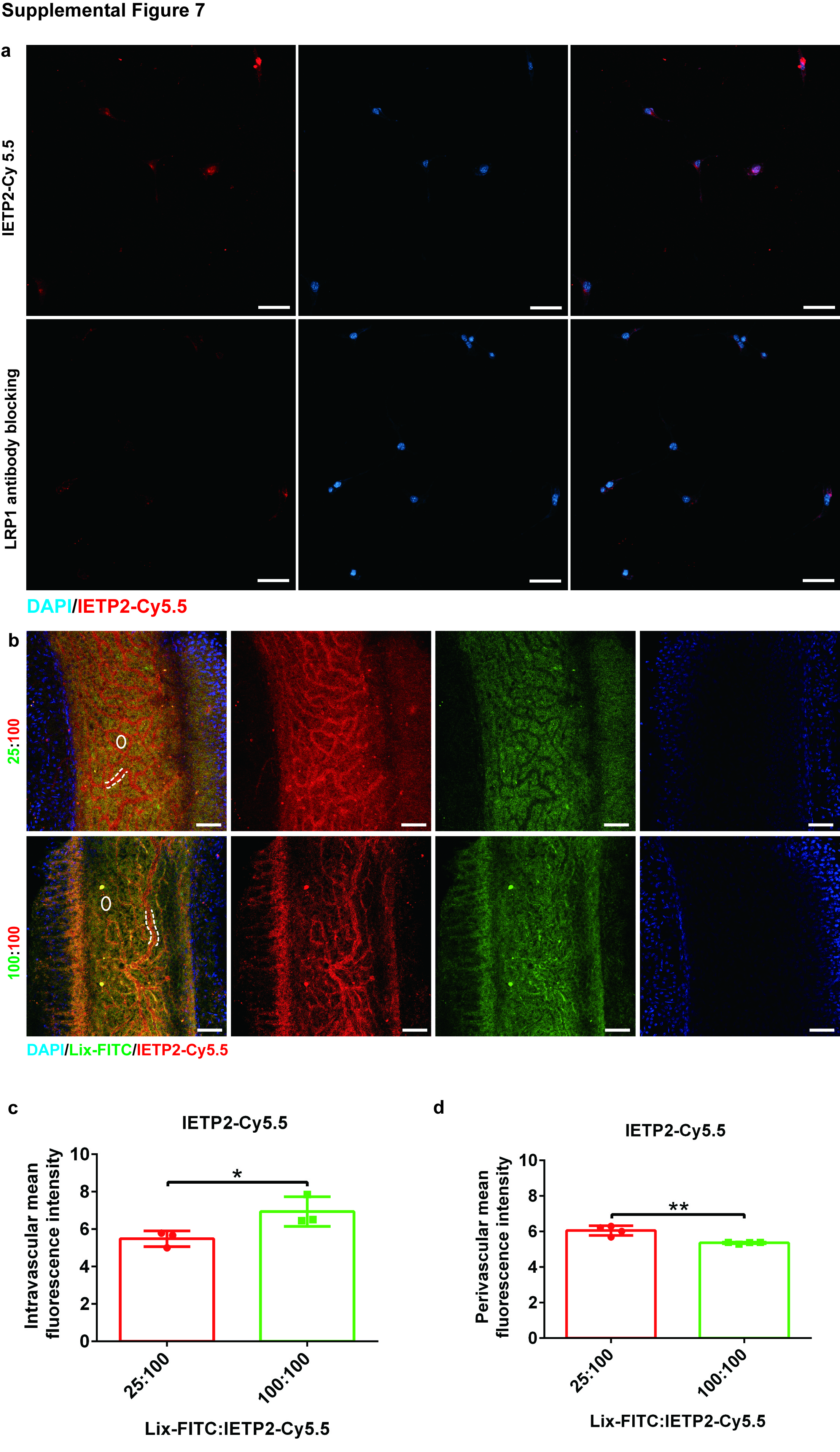


**Supplemental Figure 7 *In vitro* analysis of IETP2-Cy5.5 uptake by LRP1 antibody blocking or wild-type HEI-OC1 cells and *in vivo* competition of IETP2-Cy5.5 and Lix-FITC.**

**a.** Immunofluorescence images of LRP1-antibody-preblocked (3 h) or unblocked HEI-OC1 cells incubated with 1 μL of IETP2-Cy5.5 (3 mg/mL) for 45 min. **b.** Immunofluorescence images of the SV of mouse cochleae after intravenous injection of IETP2-Cy5.5 and its competitive ligand Lix-FITC at different volume ratios, 100:25 and 100:100 µL (DAPI: blue; Lix-FITC: green; IETP2-Cy5.5: red). * p < 0.05, ** p < 0.01. The intravascular (the dotted line indicates the area) and perivascular (the circle indicates the area) mean fluorescence intensities (three randomly selected independent regions) are analyzed in **c** and **d**, respectively.


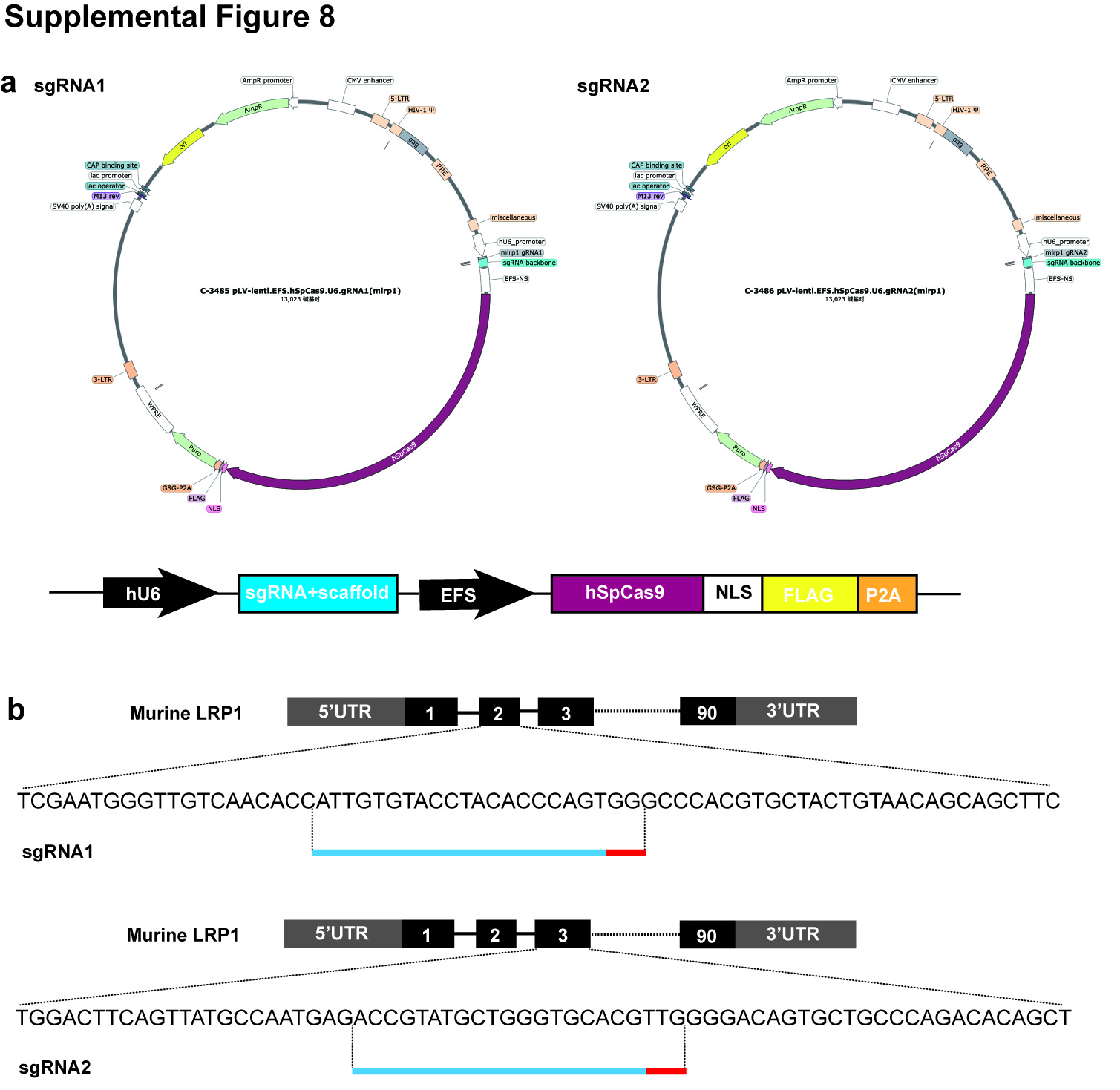


**Supplemental Figure 8 Plasmid information encoding lentivirus and gRNA sequences (related to Figure 3).**

**a**. Plasmid sequence-encoding lentivirus containing Cas9, gRNA1 and gRNA2. **b**. gRNA1 sequence targeting Exon 2, gRNA2 sequence targeting Exon 3.


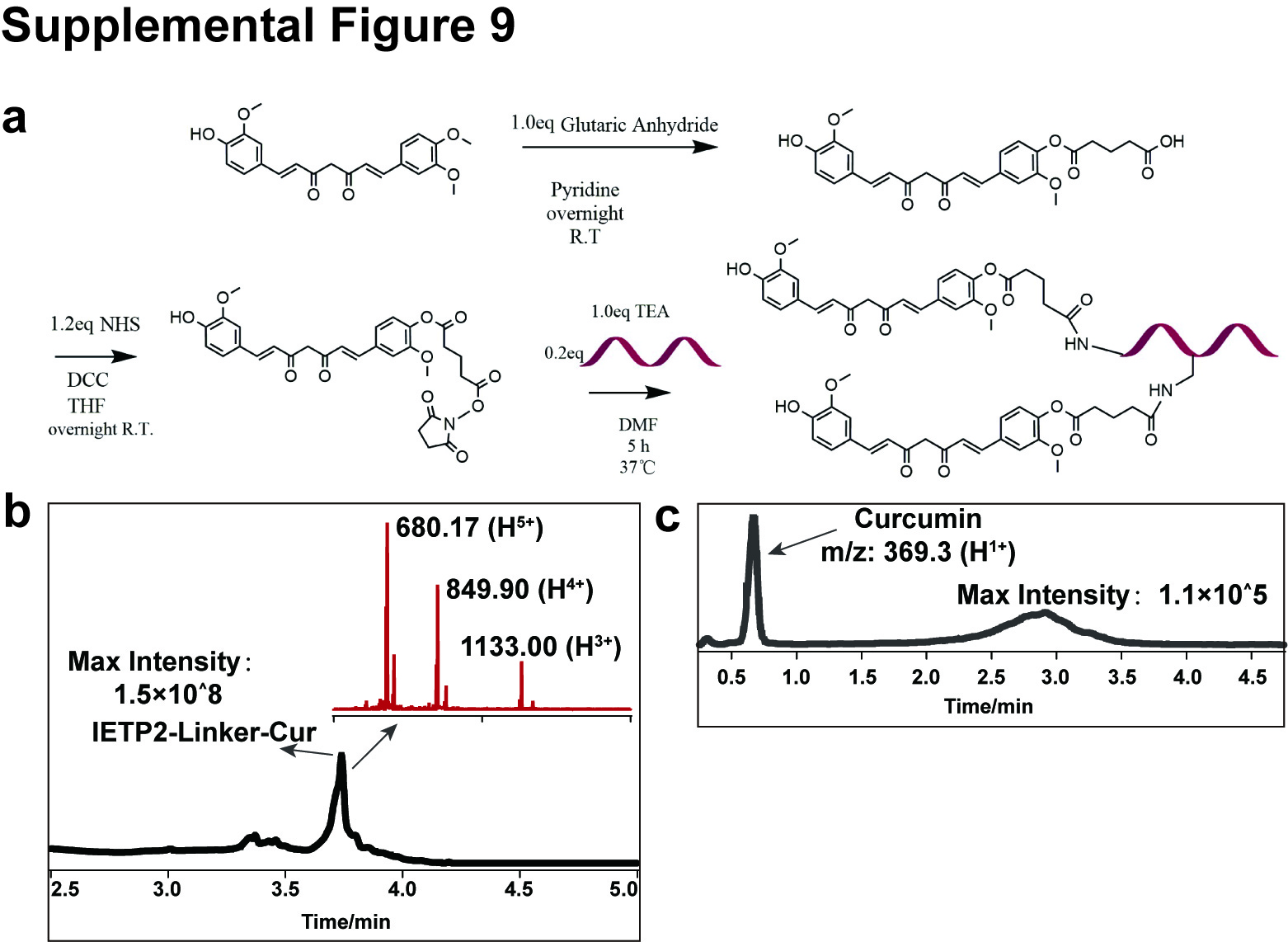


**Supplemental Figure 9 The synthesis procedure and LC–MS validation of IETP2-Linker-Cur**

**a.** Synthesis procedures for IETP2-Linker-Cur. **b.** LC–MS validation of the synthesized IETP2-Linker-Cur. **c.** Signal response of Cur in cochlear lymphatic fluid by detection of the corresponding cation peak.


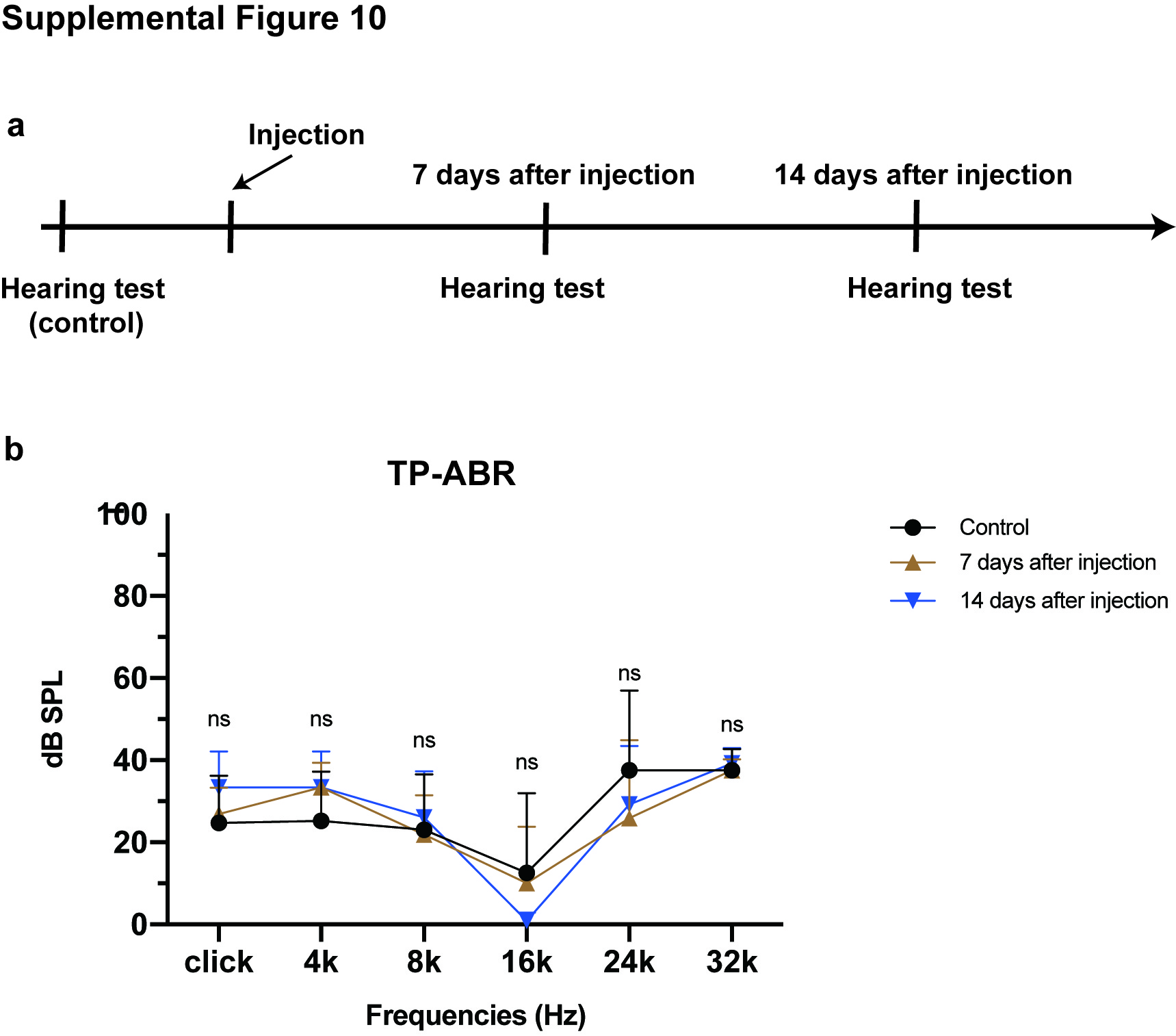


**Supplemental Figure 10 Evaluation of the ototoxicity of IETP2 injection in C57BL/6J mice.**

**a**. Schematic diagram of the schedule for injection and hearing tests. Hearing tests were taken 1 day before, 7 days after and 14 days after injection. The hearing tests taken before injection were used as an internal control for analysis. **b**. The thresholds of tone pip (TP)-ABR at different frequencies, click, 4 kHz, 8 kHz, 16 kHz, 24 kHz and 32 kHz, in the IETP2-injected mice (6 mice, 12 ears, either gender, 4 weeks old) at different time points. Average thresholds at each frequency were used to perform one-way ANOVA. (control: closed circle; 7 days after injection: brown triangle; 14 days after injection: blue).


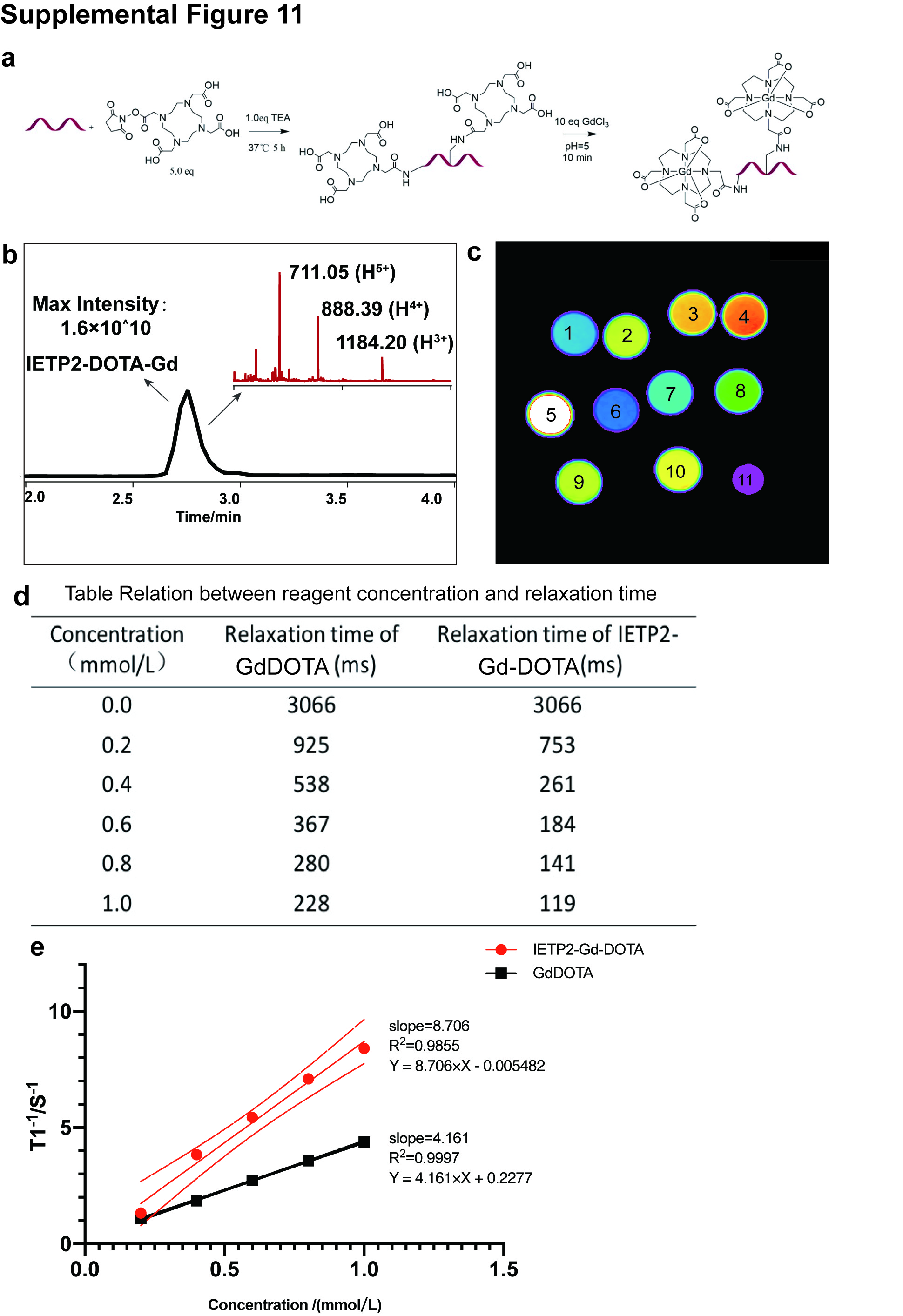


**Supplemental Figure 11 Synthesis procedure, LC–MS validation and relaxation rate measurement of IETP2-Gd-DOTA (related to Figure 5).**

**a.** Synthesis procedures for IETP2-Gd-DOTA. **b.** LC–MS validation of the synthesized IETP2-DOTA-Gd. **c.** MRI scanning results of the centrifuge tube. IETP2-Gd-DOTA numbered from 1 to 5 was 0.2 mmol/L to 1.0 mmol/L, respectively. The numbers 6-10 were 0.2 mmol/L-1.0 mmol/L GdDOTA. No. 11 is pure water. **d.** Calculation method of the relaxation rate: take the reagent concentration as the abscissa and 1000 divided by the relaxation time (ms) as the ordinate to make the relaxation property curve and measure the relaxation rate. **e.** The red curve is the IETP2-Gd-DOTA relaxation curve, and the curve equation is Y= 8.706X-0.005482, R^2^=0.985. The black curve is the GdDOTA relaxation curve, and the curve equation is Y= 4.161X +0.2277, R^2^=0.9997. These results show that there is a linear relationship between concentration and relaxation time for the two substances throughout the range of concentrations. Compared with GdDOTA, IETP2-Gd-DOTA with the same concentration had better imaging properties.


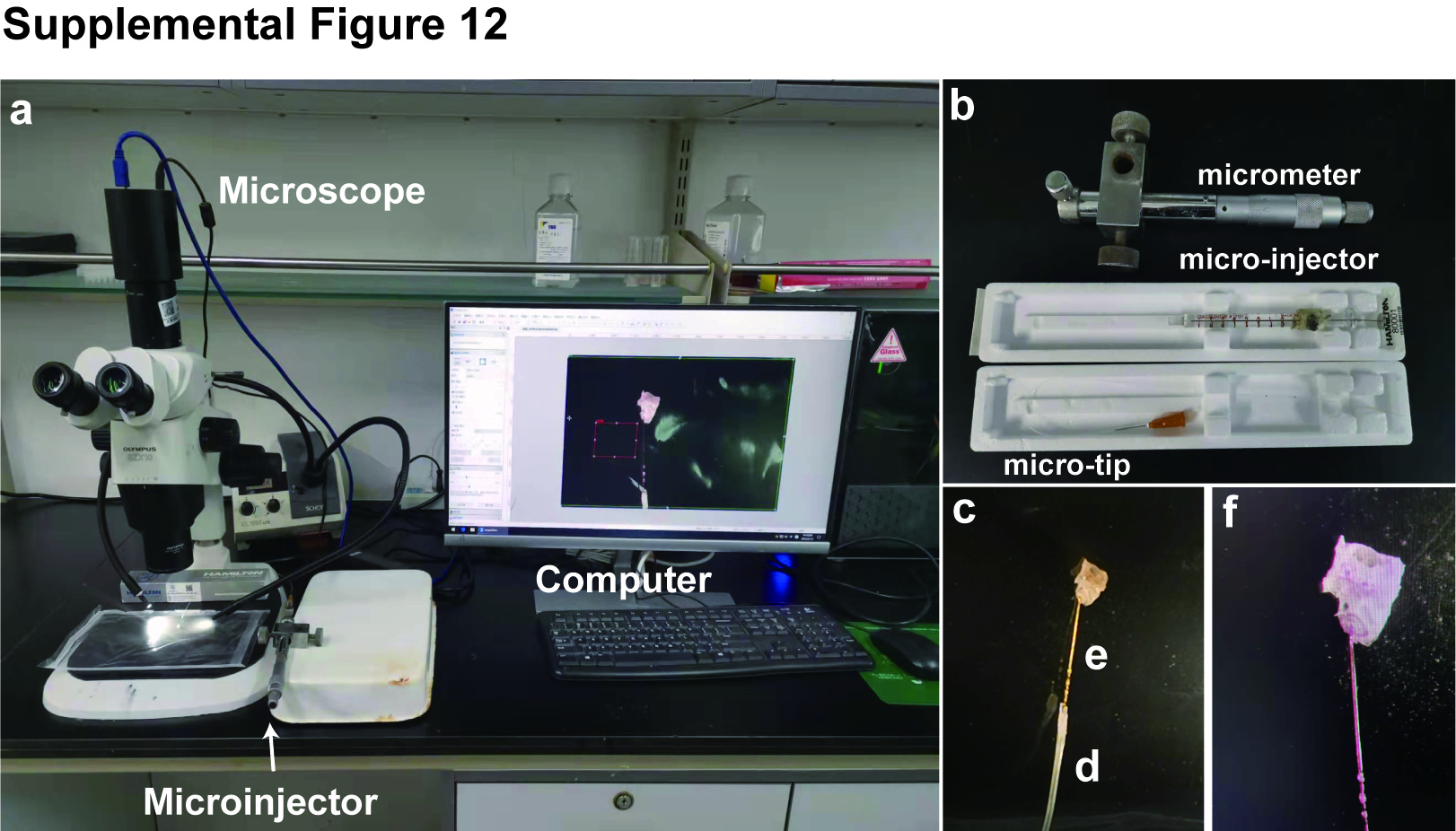


**Supplemental Figure 12 The construction of the homemade microsyringe pump.**

**a**. Overview of the microsyringe pump system. A microscope and computer were used to visualize the withdrawal. **b**. The microinjector consisted of a micrometer to control the volume (the injection volume range was 1-10 µL), a microinjector (HAMILTON 80001) and a microtip (WPI, 501656). c. d is the FEP Teflon microdialysis tubing (WPI, 505515), and e is the polyimide tubing (A-M Systems, 823400), which is connected to the Teflon tubing. The microtip connected the microinjector (b) to the Teflon microdialysis tubing (d). **e**. Tubing was placed at the apex hole, and lymphatic fluid was withdrawn using a microsyringe pump.
